# Increased Plasmatic Levels of PSA-Expressing Exosomes Distinguish Prostate Cancer Patients from Benign Prostatic Hyperplasia: A Prospective Study

**DOI:** 10.3390/cancers11101449

**Published:** 2019-09-27

**Authors:** Mariantonia Logozzi, Daniela F. Angelini, Alessandro Giuliani, Davide Mizzoni, Rossella Di Raimo, Martina Maggi, Alessandro Gentilucci, Vittorio Marzio, Stefano Salciccia, Giovanna Borsellino, Luca Battistini, Alessandro Sciarra, Stefano Fais

**Affiliations:** 1 Department of Oncology and Molecular Medicine, Istituto Superiore di Sanità, Viale Regina Elena 299, 00161 Rome, Italy; mariantonia.logozzi@iss.it (M.L.); davide.mizzoni@iss.it (D.M.); rossella.diraimo@iss.it (R.D.R.); 2 Neuroimmunology Unit, IRCCS Santa Lucia Foundation, 00179 Rome, Italy; df.angelini@hsantalucia.it (D.F.A.); g.borsellino@hsantalucia.it (G.B.); l.battistini@hsantalucia.it (L.B.); 3 Environment and Health Department Istituto Superiore di Sanità, Viale Regina Elena 299, 00161 Rome, Italy; alessandro.giuliani@iss.it; 4 Department of Urology, Policlinico Umberto I, Università La Sapienza, Viale dell’Università 33, 00161 Rome, Italy; martina.maggi@uniroma1.it (M.M.); alegenti@yahoo.com (A.G.); v.marzio89@gmail.com (V.M.); stefano.salciccia@uniroma1.it (S.S.); alessandro.sciarra@uniroma1.it (A.S.)

**Keywords:** prostate cancer (PCa), benign prostatic hyperplasia (BPH), exosomes, ELISA, nanoscale flow cytometry

## Abstract

Prostate Specific Antigen (PSA) fails to discriminate between benign prostatic hyperplasia (BPH) and Prostate Cancer (PCa), resulting in large numbers of unnecessary biopsies and missed cancer diagnoses. Nanovesicles called exosomes are directly detectable in patient plasma and here we explore the potential use of plasmatic exosomes expressing PSA (Exo-PSA) in distinguishing healthy individuals, BPH, and PCa. Exosomes were obtained from plasma samples of 80 PCa, 80 BPH, and 80 healthy donors (CTR). Nanoparticle Tracking Analysis (NTA), immunocapture-based ELISA (IC-ELISA), and nanoscale flow-cytometry (NSFC), were exploited to detect and characterize plasmatic exosomes. Statistical analysis showed that plasmatic exosomes expressing both CD81 and PSA were significantly higher in PCa as compared to both BPH and CTR, reaching 100% specificity and sensitivity in distinguishing PCa patients from healthy individuals. IC-ELISA, NSFC, and Exo-PSA consensus score (EXOMIX) showed 98% to 100% specificity and sensitivity for BPH-PCa discrimination. This study outperforms the conventional PSA test with a minimally invasive widely exploitable approach.

## 1. Introduction

Prostate cancer (PCa) is the most commonly diagnosed cancer and the second leading cause of cancer-related deaths in human males; diagnosis of PCa and subsequent treatments have high medical, psychological, and economic impact [1].

The current standard method for PCa diagnosis is transrectal ultrasound (TRUS)-guided prostate biopsy, which is mainly performed on the basis of abnormal plasmatic levels of prostate-specific antigen (PSA) [2]. However, PSA is organ- but not cancer-specific and PCa screening using a PSA–based threshold as the sole indication for prostate biopsy results in large numbers of unnecessary biopsies. Moreover, the low specificity of this test leads to high numbers of undiagnosed PCa [3].

In order to add sensitivity and specificity to PSA testing and to avoid unnecessary biopsies, several alternative approaches have been developed over the years [4,5] but none can yet be implemented for routine screening programs. To date, digital rectal examination remains a primary test for the initial diagnosis of PCa [1,2,3,4] and although serum PSA determination is used worldwide for PCa early diagnosis [6], its use has become controversial for the high number of false positives and false negatives it provides [1,3,7,8,9,10,11].

Extracellular vesicles, which include nanovesicles (30–100 nm) called exosomes, are carriers for biomolecules including proteins, lipids, and nucleic acids [12], thus representing potential source of disease biomarkers including cancer [13].

Exosomes are released in human body fluids, including plasma, sperm, and urine by a variety of cells both in physiological and pathological conditions [12]. Exosome release dramatically increases during tumorigenesis and when exposed to some micro-environmental factors such as low extracellular pH [14], independently from the tumor histotype [15]. Tumor exosomes circulate in the body, shuttling bio-markers including coding and non-coding RNAs [12,14,16,17]. Recently, liquid biopsies have emerged as valid alternatives to standard tumor biopsies. Tumor-derived exosomes, released or spilled-over into the body fluids, may well represent key prototypes as liquid biopsies, with both diagnostic and prognostic applications.

This study aimed at evaluating the clinical relevance of plasmatic exosomes expressing PSA in a large cohort of prostate cancer (PCa) and benign prostatic hyperplasia (BPH) patients, and in healthy subjects. The experimental protocol included the use of both nanoscale flow-cytometry and immunocapture-based ELISA for extracellular vesicles characterization and quantification, and nanoparticle tracking analysis (NTA) for quality control of plasmatic samples after the ultracentrifugation. These tests have been performed in up to 240 plasma samples deriving from either PCa and BPH and healthy individuals. In each individual, the levels of of plasmatic exosomes expressing PSA were compared to the standard serum PSA, but in healthy controls that were all males under 30. The results have shown that plasmatic exosomes expressing PSA distinguished between PCa patients and both BPH and healthy individuals, with both sensitivity and specificity significantly higher than serum PSA with all the exploited tests.

## 2. Results

### 2.1. Identification and Characterization of Exosomes from Patients and Controls

The NTA provided a quality control for the exosome preparations, showing that the distribution profiles of plasmatic exosomes obtained from both healthy donors and BPH patients were more heterogeneous in size and distribution [18]; while the profile of exosomes obtained from the plasma of PCa patients was homogeneous in terms of size and distribution (Supplementary Figure S1A). The investigation on the importance of exosomes number and size, as assessed by NTA, deserves an entirely dedicated study in which exosome levels before and after surgery have to be investigated as well, in order to understand better whether both these parameters are indeed due to the presence of the malignant tumors, as shown for other cancers [19]. Exosomal preparations from CTR, BPH and PCa plasma were further characterized by Western blot analysis for housekeeping markers of exosomes, Tsg 101 and CD81 (Supplementary Figure S1B).

Each plasma sample underwent both immunocapture-based ELISA (IC-ELISA) and NanoScale Flow Cytometry (NSFC), In both the analyses an antibody specific for a typical exosome antigen (CD81) was exploited to identify exosomes within the pool of extracellular vesicles, and an antibody for PSA was used for the detection of plasmatic exosomes expressing PSA. Using this approach, we compared the levels of PSA-expressing exosomes (Exo-PSA) between patients with PCa and patients with BPH and CTR. In a separate set of statistical analysis, we related the levels of serum PSA (S-PSA) in PCa and BPH to Exo-PSA as assessed by either IC-ELISA, NSFC, or both. All samples that did not meet the quality requirements (emolysis, hyperlipidemia, and insufficient volume) and typical characteristics of exosomes (size, distribution, and number of the exosomes analyzed by NTA) were excluded from further analysis (i.e., 10 PCa, 9 BPH, and 10 CTR were excluded from IC-ELISA, and 13 PCa, 18 BPH, and 27 CTR were not analyzed by NFSC).

### 2.2. Analysis of the PSA-Expressing Exosomes in the Plasma of Either PCa or BPH Patients or Healthy Donors

#### 2.2.1. IC-ELISA

Clinical evaluation of the plasmatic levels of exosomes expressing PSA (from 1 mL of plasma) was performed by IC-ELISA on plasma from BPH, PCa, and healthy individuals. Exosome UC preparations were seeded on anti-CD81-covered plates and then an anti-PSA antibody was added.

**Figure 1:**
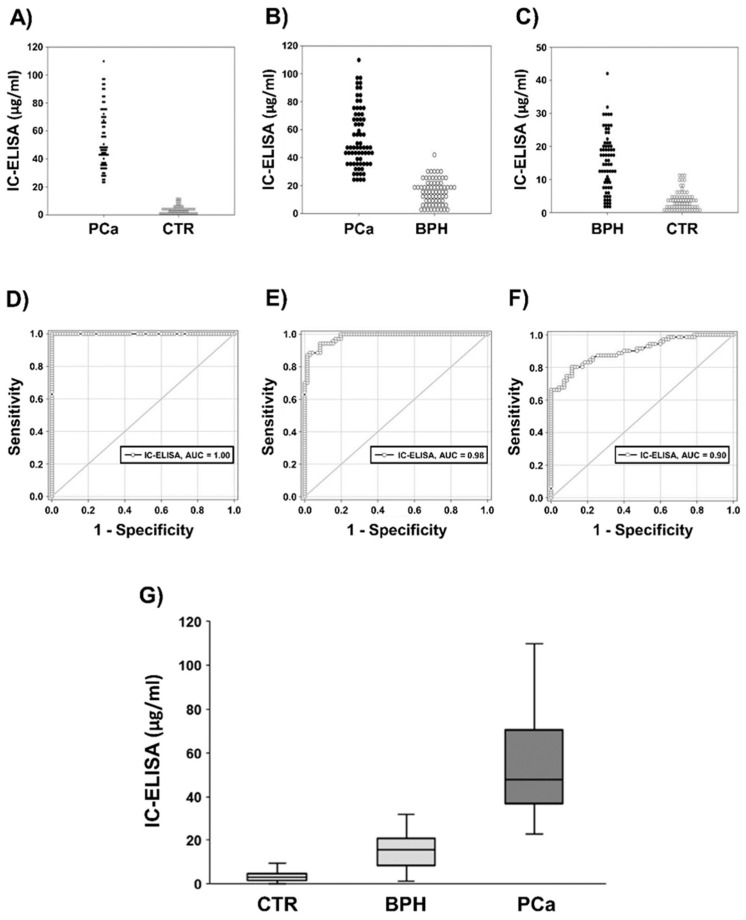
Distribution and receiving operating characteristics (ROC) curve of plasmatic exosomes expressing PSA (Exo-PSA) from healthy donors (CTR), benign prostatic hyperplasia (BPH), and prostate cancer (PCa) plasma samples analyzed with immunocapture-based ELISA (IC-ELISA). (**A**) Distribution between PCa and CTR. (**B**) Distribution between PCa and BPH. (**C**) Distribution between BPH and CTR. (**D**) ROC curve between PCa and BPH. (**E**) ROC curve between PCa and BPH. (**F**) ROC curve between BPH and CTR. (**G**) IC-ELISA distribution of CTR, BPH, and PCa included within the 25th and 75th percentiles.

Figure 1 shows the values distribution in PCa vs. CTR (Figure 1A), PCa vs. BPH (Figure 1B), and BPH vs. CTR (Figure 1C). The performance of IC-ELISA was further evaluated with receiving operating characteristics (ROC) analysis (Figure 1D–F). The data showed 100% sensitivity and specificity comparing: (1) PCa vs. CTR (Figure 1D)—AUC: 1.00, *p* < 0.001; Cut-off: µg/mL Exo-PSA = 17.07; (2) PCa vs. BPH: 98.57 sensitivity and 80.28% specificity (Figure 1E)—AUC: 0.98, *p* < 0.001; Cut-off: µg/mL Exo-PSA = 23.32; (3) BPH vs. CTR: 98.57 sensitivity and 80.28% specificity (Figure 1F)—AUC: 0.90, *p* < 0.001; Cut-off: µg/mL Exo-PSA = 23.32.

The AUC is a general estimate of the method’s discriminant power (being 1.00 the maximal value correspondent to a perfect classifier and 0.50 to a random choice). On the other hand, given the trade-off between sensitivity and specificity, the reported values of these parameters must be intended as a possible compromise among different solutions located in the top left part of the ROC plot. Intra- and inter- test variability were calculated on six replicates of the same preparation run on three different plates and were 17.39% and 33%, respectively. The analysis by the ROC curve fixed the cut-off values to 23.32 µg/mL Exo-PSA (Figure 1E), allowing to discriminate PCa from BPH patients. The graph in Figure 1G represents the distribution of PCa, BPH, and CTR included within the 25th and 75th percentiles. PSA detection through exosome quantification by IC-ELISA discriminates PCa from BPH and CTR.

#### 2.2.2. Correlation Between IC-ELISA, NSFC and Serum PSA (S-PSA)

This set of experiments clearly showed that IC-ELISA was able to measure significantly higher plasmatic levels of Exo-PSA in patients with PCa as compared to both healthy controls (100% specificity and 100% sensitivity, perfect classifier 1.00) and patients with BPH (98.57% specificity and 80.28% sensitivity, 0.98 AUC). However, we used NSFC to further support the data obtained with IC-ELISA in those samples endowed with both information (and thus limiting to PCa and BPH groups: *n* = 132).

Only in this way, we can both clarify to what extent they refer to the same latent phenomenon and devise a prognostic strategy based on a combination of different biomarkers. The derived Log-NSFC descriptor, correspondent to the logarithm of NSFC, was added to the original biomarkers in order to eliminate the extremely high variability of NSFC Exo that could bias the observed correlation structure.

Supplementary Table S1 clearly shows that the exosome-related measures are significantly correlated, thus pointing to the same biological phenomenon: Serum PSA and exosome PSA are independent of each other. This confirms the impossibility of serum PSA for discriminating PCa and BPH patients.

Principal component analysis, as applied to the above correlation structure (the principal components are the eigenvectors of the correlation matrix and point to the latent independent factors getting rid of data set variance [20,21]), gave rise to two significant principal components cumulatively explaining 83% of the total variance. Principal components are the linear combinations of original variables maximizing the total variance of the data set, and are each other orthogonal by construction; they correspond to the “latent variables” at the basis of the observed biomarker variance [20]. The Pearson correlation coefficients (loadings) between original variables and components allow for a straightforward interpretation of component meanings (Supplementary Table S2).

**Figure 2:**
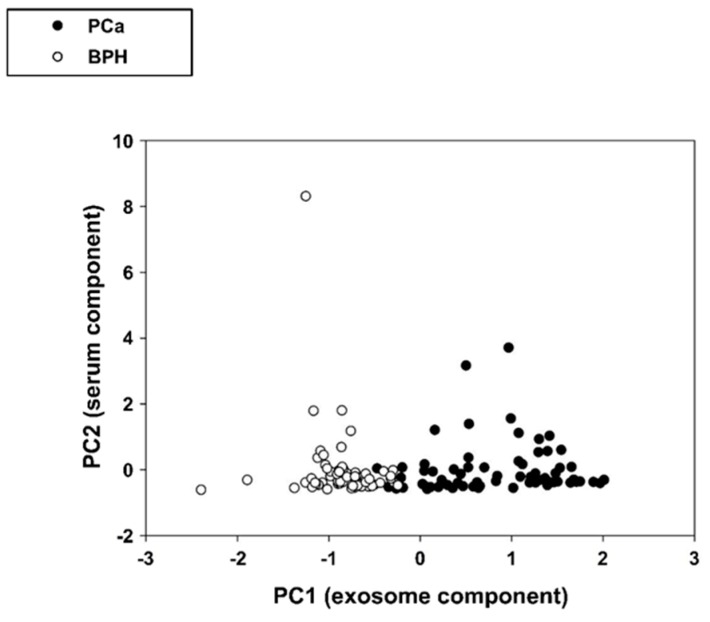
Projection (component scores) of the patients in the bi-dimensional space spanned by the two principal components (PC1 = exosome component and PC2 = serum component).

Principal component analysis is a data-driven procedure, thus the isolation of a pure cancer subset at values greater than PC1 mean value (components have by construction zero mean and unit standard deviation) was an emergent property pointing to a clear-cut discrimination of BPH and PCa patients by exosome biomarkers (S-PSA, IC-ELISA, Log-NSFC). The complete lack of discrimination ability of S-PSA was evident as well (Figure 2).

In order to generate a composite index collating the NSFC and IC-ELISA information we computed, another principal component analysis on the NSFC, Log-NSFC space (Supplementary Table S3), was carried out. In this case, the extracted components correspond to the “consensus” (PC1) and “divergent” (PC2) information carried by the two biomarkers and, by construction, correspond to a rotation of the log-NSFC/IC-ELISA space explaining the total initial information.

**Figure 3:**
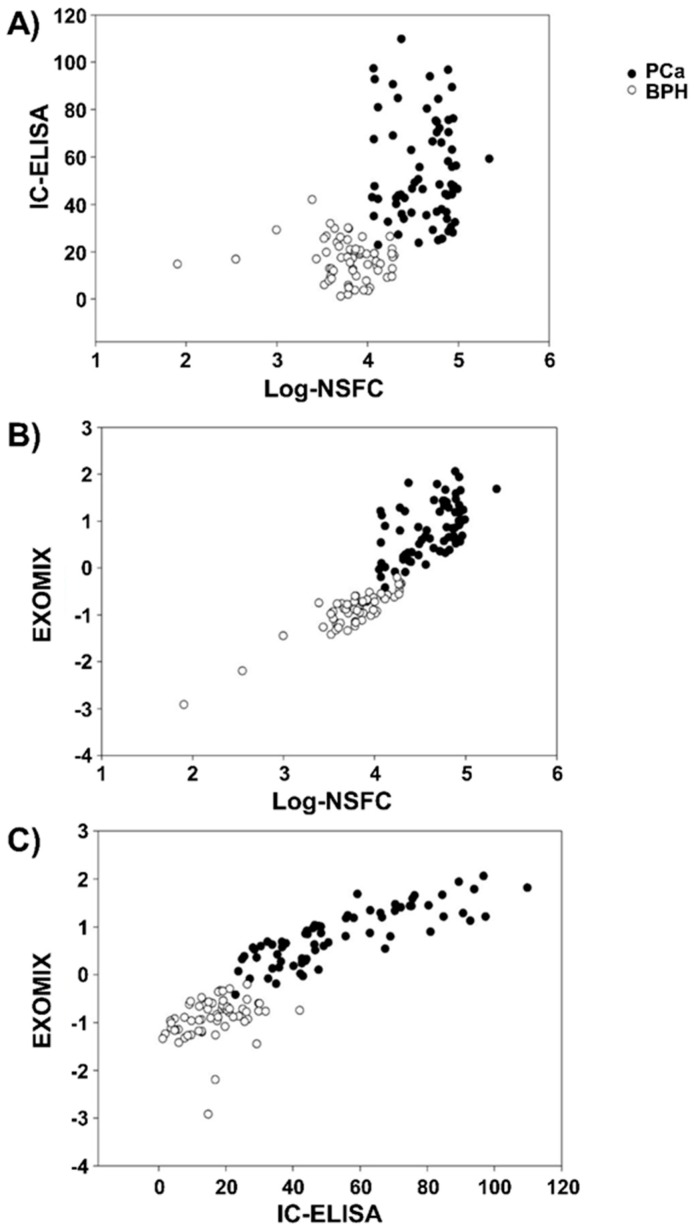
Nanoscale flow-cytometry (NSFC)/IC-ELISA original plane (**A**) together with the relations between the combined score (EXOMIX) with the exosome biomarkers (**B**,**C**).

We extracted the first principal component scores (EXOMIX) to perform a ROC analysis of the combined NSFC/ELISA information together with the original biomarkers. The top panel (Figure 3A) highlights how the convergence between NSFC and IC-ELISA approaches only holds at the “gross scale” of PCa/BPH discrimination (BPH patients occupy the left/bottom part of the graph while PCa group distributes in the right/top quadrants). On the contrary, the “within-group” variance of the two variables is largely independent (intra class Pearson correlation r = 0.05 (NS) and r = –0.14(NS) for PCa and BPH groups, respectively).

This implies that the two NSFC and IC-ELISA approaches are put into correlation by their common relation with prostate cancer but refer to two different views of the same system, carrying different information regarding the internal variance of the two groups.

The “shared” cancer-related information carried by the two biomarkers is expressed by EXOMIX, as evident in Figure 3B,C where the variability of the two descriptors is constrained along a common main direction.

**Figure 4:**
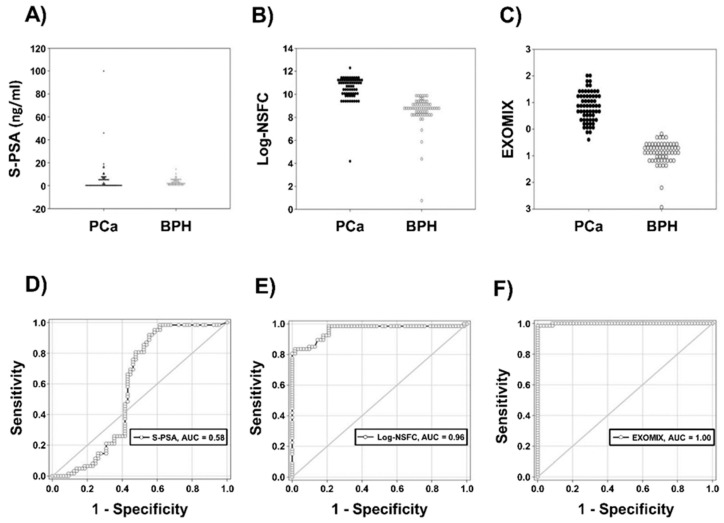
Distribution and ROC curve of S-PSA, Log-NSFC and EXOMIX for the discrimination between PCa and BPH. (**A**) Distribution of S-PSA. (**B**) Distribution of Log-NSFC. (**C**) Distribution of EXOMIX. (**D**) ROC curve of S-PSA. (**E**) ROC curve of Log-NSFC. (**F**) ROC curve of EXOMIX.

The above results have an immediate counterpart in the relative efficiency of the different descriptors in the PCa/BPH discrimination, as evidenced by ROC analysis (Table 1 and Figure 4).

## 3. Discussion

In this study, we provide a reliable and minimally invasive clinical new tool for both the early diagnosis and the clinical follow up of prostate cancer. The methodology used was previously described in a pilot study showing that the differences in terms of plasmatic levels of Exo-PSA between cancer patients and controls were consistent with the release of Exo-PSA between human prostate cancer cells cultured at different pH conditions [14]; the previous study set up all the methodologies exploited in this clinical investigation. Here we show, through a cross-sectional clinical trial, that Exo-PSA levels are significantly higher in the plasma obtained from PCa patients when compared to the levels in plasma of both healthy donors and patients with BPH. Statistical analysis showed that IC-ELISA alone reached 98.57 sensitivity and 80.28% specificity in discriminating PCa from BPH. The combination of the data obtained with both IC-ELISA and NFSC determined a rise in sensitivity to 96% and of specificity to 100%. Interestingly, with this method it is also possible to discriminate BPH patients from healthy controls.

These results represent a breakthrough in the clinical management of PCa with a minimally invasive test; moreover, our results provide the means to perform screening for prostate cancer in younger males (under 40). While exosomes can be detected in the urine of PCa patients [22,23], the data shown in this study was obtained in plasma and is therefore comparable to serum PSA measurements.

With these results we provide a novel tool of extraordinary sensitivity for the clinical approach to prostate cancer secondary prevention, with a method that can be easily adopted by clinical laboratories worldwide, possibly avoiding some sad clinical outcomes [24,25].

## 4. Materials and Methods

### 4.1. Population

The review board of each participating institution approved the trial, which was conducted in accordance with the current International Conference on Harmonisation guidelines for Good Clinical Practice and the principles of the Declaration of Helsinki. The study was approved by the Istituto Superiore di Sanità Ethics Committee on 18/04/2017 (Rif. Prot. PRE-275/17). All the patients provided written informed consent.

All the authors assume responsibility for the completeness and accuracy of the data and analyses and for the fidelity of the trial to the protocol, available with the Supplementary Materials. All the authors had full access to the data, drafted the manuscript, reviewed and approved the manuscript before submission, and made the decision to submit the manuscript for publication. No sponsor provided funding for the study.

Eligible cases were divided in 3 groups: Control cases (CTR), benign prostatic hyperplasia cases (BPH) and prostate cancer cases (PCa). All cases were consecutively included in the study as out-patients referred to Department of Urology on the basis of the inclusion criteria. Patients were correctly informed, accepted to be included in the study, and signed an informed consensus prior to each procedure. Human plasma samples were collected from EDTA-treated whole blood, 5 mL into BD Vacutainer^®^ K3-EDTA-coated collection tubes (Beckton Dickinson, Franklin Lakes, NJ, USA), from department of Urological Sciences, Policlinico Umberto I, Sapienza University of Rome, Italy. Once collected, the samples were labeled by the clinical center with an identification code and were manipulated anonymously and blinded in the testing phase with the code assigned by the clinical center.

This is an experimental observational clinical research study in which no additional and/or administered drug tests and/or modified therapy are performed.

CTR. The control group consisted of 80 male individuals consecutively referred to our department with the following inclusion criteria: Age from 18 to 39 years; no clinical evidence of BPH or PCa (digital rectal examination (DRE) and ultrasonography (US)); prostate volume less than 30 cc; total PSA level less than 1.4 ng/mL; no familiarity for PCa; no therapies that can influence PSA determination.

BPH. The BPH group consisted of 80 male individuals consecutively referred to our department with the following inclusion criteria: Age from 45 to 75 years, histologically confirmed diagnosis of BPH; no clinical and pathological evidence of PCa; no therapies that can influence PSA determination (e.g., 5 alpha reductase inhibitors)

PCa. The PCa group consisted of 80 male individuals consecutively referred to our department with a histologically confirmed diagnosis of prostate adenocarcinoma (prostate biopsy). None of cases were submitted to androgen deprivation therapies or other therapies that can influence PSA determination. All cases were stratified in risk classes (EAU classification) on the basis of total PSA levels, Gleason score, and clinical stage.

### 4.2. Preparation of Exosomes from Plasma of Patients and Controls

To obtain plasma from blood samples, EDTA-treated blood from PCa patients, BPH patients, and CTR were centrifuged at 400× *g* for 20 min. Plasma was then collected and stored at −80 °C until analysis. Upon thawing, 1 mL of plasma underwent the centrifugal procedure as previously described [18,26] in order to pellet exosomes. Plasma samples were centrifuged for 1 h 30 min at 110,000× *g* using a Fiberlite™ F50L-24 × 1.5 Fixed-Angle Rotor, K-Factor: 33 (Thermo Fisher Scientific, Waltham, MA, USA) in the Sorvall WX Ultracentrifuge Series (Thermo Fisher Scientific).

### 4.3. Assays for Plasmatic Exosomes Characterization and Quantification

#### 4.3.1. Nanoparticle Tracking Analysis

Nanoparticle Tracking Analysis (NTA) from Malvern (NanoSight NS300, Worcestershire, UK) was used for the measurement of size distribution and concentration of extracellular vesicles samples in liquid suspension [14]. Five videos of typically 60 s duration were taken. Data were analyzed using the NTA 3.0 software (Malvern Instruments) which was optimized to first identify and then track each particle on a frame-by-frame basis. The Brownian motion of each particle was tracked using the Stokes–Einstein equation: D° = kT/6πηr, where D° is the diffusion coefficient, kT/6πηr = f0 is the frictional coefficient of the particle, for the special case of a spherical particle of radius r moving with uniform velocity in a continuous fluid of viscosity η, k is Boltzmann’s constant, and T is the absolute temperature.

#### 4.3.2. Western Blot

For each group (CTR, BPH, PCa) 4 mL of plasma was pooled and Size Exclusion Chromatography (SEC) was performed for the isolation of plasma-derived exosomes, as described previously [27]. Exosomes from plasma of CTR, BPH and PCa patients were lysed in CHAPS buffer 1 × containing Tris 10 mM pH 7.4, MgCl2 1 mM, ethyleneglycoltetraacetic acid (EGTA) 1 mM, CHAPS 0.5%, glycerol 10%, phenylmethylsulfonyl fluoride (PMSF) 1 mM and protease inhibitor cocktail (1 µg/mL leupeptin, 1 µg/mL pepstatin A, 1 µg/mL aprotinin, and PMSF 1 mM).

Protein concentration was determined using the Bradford protein assay (Bio-Rad Laboratories, Inc, Hercules, CA, USA). Thirty micrograms of exosomal lysates were resolved on 10% acrylamide gel and transferred to a Protran BA85 nitrocellulose membrane (Schleicher & Schuell, Keene, NH, USA). Nonspecific binding sites were blocked by incubation in PBS containing 0.05% Tween 20 and 5% milk powder. Blotting was performed employing anti-Tsg101 (4A10, GeneTex, Irvine, CA, USA), and anti-CD81 (B-11, Santa Cruz Biotechnology, Dallas, TX, USA) monoclonal antibodies, for 18 h at 4 °C. After incubation with appropriate anti-mouse peroxidase-conjugated secondary antibody (IgG; Amersham Biosciences, Milan, Italy) for 1 h at room temperature, membranes were revealed by enhanced chemiluminescent (ECL) substrate (Thermo Fisher Scientific, Waltham, MA, USA).

#### 4.3.3. ELISA for PSA

96-well plates (Nunc, Milan, Italy) were coated with 4 µg/mL rabbit polyclonal anti-CD81 antibody (clone PA5-79003, Thermo Fisher Scientific, Waltham, MA, USA) in 100 µL/well of PBS and incubated overnight at 4 °C. After 3 washes with PBS, 100 µL/well of blocking solution (PBS containing 0.5% BSA) was added at room temperature for 1 h. Following 3 washes in PBS, nanovesicles purified from plasma were quantified by Bradford assay and then suspended in a final volume of 50 µL (1 µg/µL) and incubated overnight at 37 °C. After 3 washes with PBS, 4 µg/mL of a mouse anti-PSA HRP-conjugated (clone 5A6, Abcam, Cambrige, MA, USA) were added to each well and incubated for 1 h at 37 °C. After the final 3 washes with PBS, the reaction was developed with Blue POD for 15 min (Roche Applied Science, Milan, Italy), and blocked with 4N H_2_SO_4_ stop solution. Optical densities were recorded at 450 nm. A PSA calibration curve was previously described [14]. The PSA calibration curve allowed to convert the optical densities of each sample into micrograms of Exo-PSA.

#### 4.3.4. Flow Cytometry Analysis of Exosomes

Exosomes purified from plasma were diluted in PBS in a final volume of 50 µL. Anti-human CD81 allophycocyanin (APC) conjugated (Beckman Coulter; Brea, CA, USA) and anti-human PSA fluorescein (FITC) conjugated (clone 5A6, Abcam) or anti IgG2a APC and IgG1 FITC (Beckman Coulter) were added to the exosome preparation at optimal pre-titered concentrations and left for 20 min at RT. 500 µL of PBS were added to samples before the acquisition on the CytoFLEX flow cytometer (Beckman Coulter). The cytometer was calibrated as previously described [14].

### 4.4. Statistical Analysis

The discriminant power of the different tests was assessed by Receiver Operating Characteristic (ROC) curves, allowing the estimation of both the average discriminant ability of the test (area under the curve, AUC) and the selection of cut-off thresholds maximizing sensitivity (percentage of correctly diagnosed cancer patients) and specificity (percentage of correctly diagnosed hypertrophy patients) [28].

Mutual correlations among tests and the generation of a consensus score between ELISA and NSFC tests (EXOMIX) were analyzed by means of the Pearson correlation and principal component analysis [20].

## 5. Conclusions

This study shows that Exo-PSA levels discriminate PCa from BPH patients and healthy controls, outperforming the conventional PSA test. The novelty and strength of this approach reside in its high specificity and fine sensitivity which enable for the first time the discrimination between PCa and BPH patients, supporting its use not only as a screening test for early PCa diagnosis but also for the follow up of patients undergoing surgery. Crucially, this test is also minimally invasive and can be widely exploited in clinical laboratories.

## Figures and Tables

**Table 1:** ROC Analysis of Different Methods.

Biomarker	ROC Area (*p*)	Sensitivity	Specificity
S-PSA	0.582 (NS)	0.76	0.54
IC-ELISA	0.982 (*p* < 0.001)	0.98	0.80
Log-NSFC	0.960 (*p* < 0.001)	0.98	0.79
EXOMIX	0.999 (*p* < 0.001)	0.96	1.00

The Areas Under ROC Curves, a random prediction corresponds to an area of 0.5, while a unit area implies a maximal prediction power, *p*-values indicate the departure from randomness. Sensitivity and Specificity refer to cut-off values in the “optimality” range for all the four approaches.

## Supplementary Materials

The following are available online at https://www.mdpi.com/2072-6694/11/10/1449/s1, Figure S1. Quality control for the exosome preparations by NTA and protein characterization by western blot analysis for housekeeping markers of exosomes, Table S1: Descriptive statistics together with the pairwise Pearson correlation coefficients between the different biomarkers (S-PSA, IC-ELISA, Log-NSFC), Table S2: Projection (component scores) of the patients in the bi-dimensional space spanned by the two principal components, Table S3: Projection (component scores) of the patients in the bi-dimensional space spanned by the two principal components and, by construction, correspond to a rotation of the Log-NSFC/IC-ELISA space explaining the total initial information. This has been provided by the authors to give readers additional information about their work.

## References

[1] Sciarra A., Gentilucci A., Salciccia S., Von Heland M., Ricciuti G.P., Marzio V., Pierella F., Musio D., Tombolini V., Frantellizzi V. (2018). Psychological and functional effect of different primary treatments for prostate cancer: A comparative prospective analysis. Urol. Oncol..

[2] Mottet N., Bellmunt J., Bolla M., Briers E., Cumberbatch M.G., De Santis M., Fossati N., Gross T., Henry A.M., Joniau S. (2017). EAU-ESTRO-SIOG Guidelines on Prostate Cancer. Part 1: Screening, Diagnosis, and Local Treatment with Curative Intent. Eur. Urol..

[3] Schröder F.H., Hugosson J., Roobol M.J., Tammela T.L.J., Ciatto S., Nelen V., Kwiatkowski M., Lujan M., Lilja H., Zappa M. (2009). Screening and prostate-cancer mortality in a randomized European study. N. Engl. J. Med..

[4] Panebianco V., Barchetti F., Sciarra A., Ciardi A., Indino E.L., Papalia R., Gallucci M., Tombolini V., Gentile V., Catalano C. (2015). Multiparametric magnetic resonance imaging vs. standard care in men being evaluated for prostate cancer: A randomized study. Urol. Oncol..

[5] Kasivisvanathan V., Emberton M., Moore C.M. (2018). MRI-Targeted Biopsy for Prostate-Cancer Diagnosis. N. Engl. J. Med..

[6] Catalona W.J., Smith D.S., Ratliff T.L., Dodds K.M., Coplen D.E., Yuan J.J., Petros J.A., Andriole G.L. (1991). Measurement of prostate-specific antigen in serum as a screening test for prostate cancer. N. Engl. J. Med..

[7] Andriole G.L., Crawford E.D., Grubb R.L., Buys S.S., Chia D., Church T.R., Fouad M.N., Gelmann E.P., Kvale P.A., Reding D.J. (2009). Mortality results from a randomized prostate-cancer screening trial. N. Engl. J. Med..

[8] Hoffman R.M. (2011). Clinical practice. Screening for prostate cancer. N. Engl. J. Med..

[9] Sturgeon C.M., Duffy M.J., Stenman U.-H., Lilja H., Brünner N., Chan D.W., Babaian R., Bast R.C., Dowell B., Esteva F.J. (2008). National Academy of Clinical Biochemistry laboratory medicine practice guidelines for use of tumor markers in testicular, prostate, colorectal, breast, and ovarian cancers. Clin. Chem..

[10] Vickers A.J., Sjoberg D.D., Ulmert D., Vertosick E., Roobol M.J., Thompson I., Heijnsdijk E.A.M., De Koning H., Atoria-Swartz C., Scardino P.T. (2014). Empirical estimates of prostate cancer overdiagnosis by age and prostate-specific antigen. BMC Med..

[11] Steuber T., O’Brien M.F., Lilja H. (2008). Serum markers for prostate cancer: A rational approach to the literature. Eur. Urol..

[12] Fais S., O’Driscoll L., Borras F.E., Buzas E., Camussi G., Cappello F., Carvalho J., Cordeiro da Silva A., Del Portillo H., El Andaloussi S. (2016). Evidence-Based Clinical Use of Nanoscale Extracellular Vesicles in Nanomedicine. ACS Nano..

[13] Shah R., Patel T., Freedman J.E. (2018). Circulating Extracellular Vesicles in Human Disease. N. Engl. J. Med..

[14] Logozzi M., Angelini D.F., Iessi E., Mizzoni D., Di Raimo R., Federici C., Lugini L., Borsellino G., Gentilucci A., Pierella F. (2017). Increased PSA expression on prostate cancer exosomes in in vitro condition and in cancer patients. Cancer Lett..

[15] Logozzi M., Mizzoni D., Angelini D.F., Di Raimo R., Falchi M., Battistini L., Fais S. (2018). Microenvironmental pH and Exosome Levels Interplay in Human Cancer Cell Lines of Different Histotypes. Cancers.

[16] Al-Nedawi K., Meehan B., Micallef J., Lhotak V., May L., Guha A., Rak J. (2008). Intercellular transfer of the oncogenic receptor EGFRvIII by microvesicles derived from tumour cells. Nat. Cell Biol..

[17] Valadi H., Ekström K., Bossios A., Sjöstrand M., Lee J.J., Lötvall J.O. (2007). Exosome-mediated transfer of mRNAs and microRNAs is a novel mechanism of genetic exchange between cells. Nat. Cell Biol..

[18] Théry C., Witwer K.W., Aikawa E., Alcaraz M.J., Anderson J.D., Andriantsitohaina R., Antoniou A., Arab T., Archer F., Atkin-Smith G.K. (2018). Minimal information for studies of extracellular vesicles 2018 (MISEV2018): A position statement of the International Society for Extracellular Vesicles and update of the MISEV2014 guidelines. J. Extracell Vesicles.

[19] Rodríguez Zorrilla S., Pérez-Sayans M., Fais S., Logozzi M., Gallas Torreira M., García García A. (2019). A Pilot Clinical Study on the Prognostic Relevance of Plasmatic Exosomes Levels in Oral Squamous Cell Carcinoma Patients. Cancers.

[20] Giuliani A. (2017). The application of principal component analysis to drug discovery and biomedical data. Drug Discov. Today.

[21] Preisendorfer R.W. (1988). Principal Component Analysis in Meteorology and Oceanography.

[22] Dijkstra S., Birker I.L., Smit F.P., Leyten G.H.J.M., de Reijke T.M., van Oort I.M., Mulders P.F.A., Jannink S.A., Schalken J.A. (2014). Prostate cancer biomarker profiles in urinary sediments and exosomes. J. Urol..

[23] Mitchell P.J., Welton J., Staffurth J., Court J., Mason M.D., Tabi Z., Clayton A. (2009). Can urinary exosomes act as treatment response markers in prostate cancer?. J. Transl. Med..

[24] Draisma G., Etzioni R., Tsodikov A., Mariotto A., Wever E., Gulati R., Feuer E., de Koning H. (2009). Lead time and overdiagnosis in prostate-specific antigen screening: Importance of methods and context. J. Natl. Cancer Inst..

[25] Fall K., Fang F., Mucci L.A., Ye W., Andrén O., Johansson J.-E., Andersson S.-O., Sparén P., Klein G., Stampfer M. (2009). Immediate risk for cardiovascular events and suicide following a prostate cancer diagnosis: Prospective cohort study. PLoS Med..

[26] Caby M.-P., Lankar D., Vincendeau-Scherrer C., Raposo G., Bonnerot C. (2005). Exosomal-like vesicles are present in human blood plasma. Int. Immunol..

[27] Baranyai T., Herczeg K., Onódi Z., Voszka I., Módos K., Marton N., Nagy G., Mäger I., Wood M.J., El Andaloussi S. (2015). Isolation of Exosomes from Blood Plasma: Qualitative and Quantitative Comparison of Ultracentrifugation and Size Exclusion Chromatography Methods. PLoS ONE.

[28] Hanley J.A., McNeil B.J. (1982). The meaning and use of the area under a receiver operating characteristic (ROC) curve. Radiology.

