# Supplementary material for: Increased Plasmatic Levels of PSA-Expressing Exosomes Distinguish Prostate Cancer Patients from Benign Prostatic Hyperplasia: A Prospective Study
Source: Cancers (Basel). 2019 Sep 27;11(10):1449. doi: 10.3390/cancers11101449 (PMC6826376; doi:10.3390/cancers11101449)
Supplement: Supplementary file 1 [file cancers-11-01449-s001.pdf]

# Supplementary Materials: Increased Plasmatic Levels of PSA-Expressing Exosomes Distinguish Prostate Cancer Patients from Benign Prostatic Hyperplasia: a Prospective Study

**Mariantonia Logozzi, Daniela F. Angelini, Alessandro Giuliani, Davide Mizzoni, Rossella Di Raimo, Martina Maggi, Alessandro Gentilucci, Vittorio Marzio, Stefano Salciccia, Giovanna Borsellino, Luca Battistini, Alessandro Sciarra and Stefano Fais**

## Supplementary Figure S1

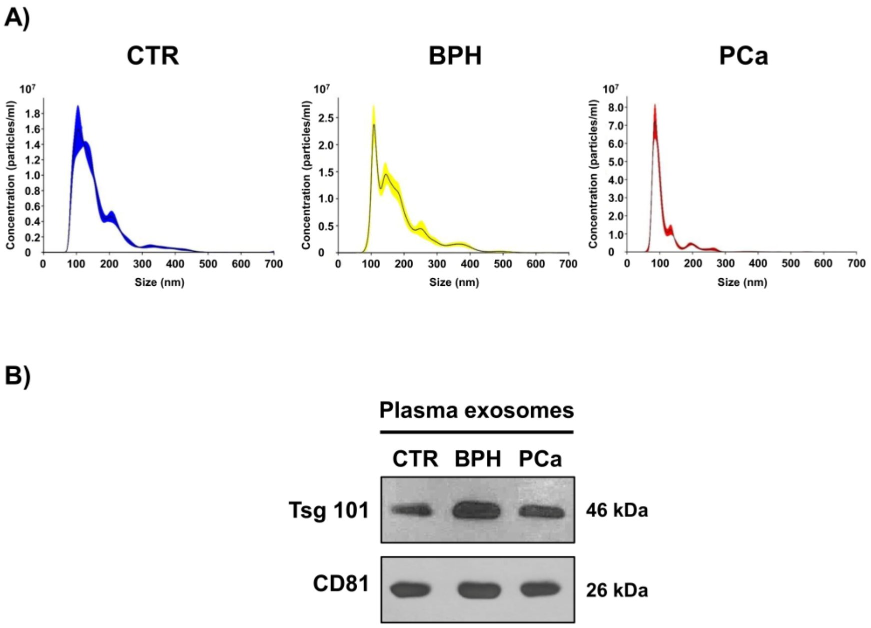


**Figure S1.** Quality control for the exosome preparations by NTA and protein characterization by western blot analysis for housekeeping markers of exosomes. A) Distribution of exosomes purified from the plasma of CTR, BPH and PCa through NTA. One representative NTA plot showing the size, concentration and distribution of the exosomes preparations obtained from CTR, BPH and PCa plasma samples. B) Western blot analyses of tumor susceptibility gene 101 (Tsg 101, 46 kDa), and cluster of differentiation 81 (CD81, 26 kDa) proteins, performed in total protein extracts of exosomes purified from the plasma of CTR, BPH and PCa patients.

**Table S1.** Descriptive statistics together with the pairwise Pearson correlation coefficients between the different biomarkers (S-PSA, IC-ELISA, Log-NSFC) are reported.

| Descriptive statistics | | | |
|---|---|---|---|
| | **Mean** | **Median** | **Std. Dev.** |
| **NSFC** | 31290.6 | 17196 | 33515.6 |
| **S-PSA** | 5.96 | 2.77 | 11.2 |
| **IC-ELISA** | 36.125 | 29.506 | 25.16 |
| **Log-NSFC** | 9.73018 | 9.75 | 1.25 |

| Correlation Matrix (Pearson r) | | | | |
|---|---|---|---|---|
| | **NSFC** | **S-PSA** | **IC-ELISA** | **Log-NSFC** |
| **NSFC** | 1 | −0.033 | **0.485** | **0.831** |
| **S-PSA** | | 1 | −0.052 | −0.041 |
| **IC-ELISA** | | | 1 | **0.546** |
| **Log-NSFC** | | | | 1 |

| Descriptive statistics | | | |
|---|---|---|---|
| | **Mean** | **Median** | **Std. Dev.** |
| **NSFC** | 31290.6 | 17196 | 33515.6 |
| **S-PSA** | 5.96 | 2.77 | 11.2 |
| **IC-ELISA** | 36.125 | 29.506 | 25.16 |
| **Log-NSFC** | 9.73018 | 9.75 | 1.25 |

| Correlation Matrix (Pearson r) | | | | |
|---|---|---|---|---|
| | **NSFC** | **S-PSA** | **IC-ELISA** | **Log-NSFC** |
| **NSFC** | 1 | −0.033 | **0.485** | **0.831** |
| **S-PSA** | | 1 | −0.052 | −0.041 |
| **IC-ELISA** | | | 1 | **0.546** |
| **Log-NSFC** | | | | 1 |

Bolded values point to statistically significant correlations: it is immediate to note that, while exosome based measures (NSFC, Log-NSFC, IC-ELISA) are each other correlated, the S-PSA is completely independent from exosome measures.

**Table S2.** Projection (component scores) of the patients in the bi-dimensional space spanned by the two principal components.

| Variable | PC1 | PC2 |
|---|---|---|
| NSFC | 0.92 | 0.05 |
| S-PSA | −0.08 | 0.99 |
| IC-ELISA | 0.76 | −0.01 |
| Log-NSFC | 0.93 | 0.04 |
| % expl. variance | 57.8 | 24.9 |

The bolded values correspond to the variables more relevant for the interpretation of components: here it is evident how the first component (PC1) describes the EXO-PSA concentration while PC2 is practically coincident ($r$ = 0.99) with S-PSA, this result points to two independent latent factors as for exosome and serum PSA.

**Table S3.** Projection (component scores) of the patients in the bi-dimensional space spanned by the two principal components and, by construction, correspond to a rotation of the Log-NSFC / IC-ELISA space explaining the total initial information.

| Variable | PC1 (EXOMIX) | PC2 |
|---|---|---|
| IC-ELISA | 0.88 | 0.48 |
| Log-NSFC | 0.88 | −0.48 |
| % of expl.variance | 77.3 | 22.7 |

# Appendix A

This appendix has been provided by the authors to give readers additional information about their work.

This supplement contains the following items:

1. Original Protocol
2. Statistical Analysis Plan
3. Ethical Committee Approval

**Table of contents**


Table of contents ... 3

Study Summary ... 4

Abbreviation Term ... 4

Background ... 5

Rationale and Objectives ... 6

1. Primary endpoint ... 6
2. Secondary endpoint ... 6

Study Design ... 6

A. Study Population ... 6

1. Patients recruitment ... 6
2. Patient assignment to treatment group ... 7
3. Inclusion Criteria ... 7
4. Exclusion Criteria ... 7

B. Study Interventions ... 7

C. Duration of the patients recruitment ... 7

D. Patient Safety ... 7

E. Study scheme by site ... 8

F. Patients characteristics ... 8

G. Cost ... 9

Study Methods ... 9

A. Preparation of Exosomes from plasma of Patients and Controls ... 9

B. Assays for plasmatic Exosomes characterization and quantification ... 9

1. Nanoparticle tracking analysis ... 9
2. IC-ELISA for PSA ... 9
3. PSA calibration curve ... 10
4. Flow cytometry analysis of Exosomes ... 10

C. Statistical Analysis ... 10

D. Experimental scheme ... 11

Statistical methods ... 11

1. Simple statistics on total data set: MEANS procedure and Coefficient of variation (CV) ... 11

2. Correlation on total data set: Pearson correlation coefficient. ..... 12

3. Correlation intra-classes..... 12

4. Multidimensional analysis: S-PSA, IC-ELISA, Log-NSFC..... 12

Initial factorial method: Main components ..... 12

5. Construction of an explicit model of calculation of Factor1 (EXOMIX = consensus between the two estimation PSA methods of the Exo-PSA) ..... 12

6. Discriminating Analysis: IC-ELISA combined with Log-NSFC (EXOMIX)..... 13

7. Three-class discriminant analysis (PCa, BPH, CTR) with Log-NSFC..... 13

8. Inferential statistics: the TTEST procedure ..... 14

ROC Curves ..... 20

1. ROC curve IC-ELISA. ..... 20

A. ROC Curve IC-ELISA, PCa vs. CTR ..... 20

B. ROC Curve IC-ELISA, PCa vs. BPH..... 22

C. ROC Curve IC-ELISA, BPH vs. CTR..... 24

2. ROC Curve S-PSA, PCa vs. BPH..... 27

3. ROC Curve log-NSFC, PCa vs. BPH ..... 28

4. ROC Curve EXOMIX. PCA vs. BPH ..... 30

References..... 32

Ethical Committee Approval ..... 34

**Study Summary**

It is a prospective experimental clinical research study developing from a previous study (Logozzi et al, 2017) which showed an increase in PSA expression on exosomes purified from both prostate cancer cell lines (LNCaP) and plasma of patients with benign prostatic hypertrophy and prostate cancer (*n* = 10). It is a study that is based on the clinical practice practiced in the Department of Urology, UOC A Director Prof A. Sciarra) which does not provide for its implementation nor additional clinical and/or diagnostic examinations for patients or treatment or modification of an ongoing treatment. No funding or additional costs are foreseen for the realization of this project.

**Keywords:** PCa; BPH; Exo-PSA; Cut-off; Exosomes; ELISA; Nanoscale Flow Cytometry

---

| **Abbreviation** | **Term** |
|---|---|
| CTR | healty donor ConTRols |
| PCa | Prostatic cancer |
| BPH | Benign Prostatic Hyperplasia |
| PSA | Prostate-Specific Antigen |
| Exo-PSA | Exosome-associated PSA |
| S-PSA | Serum PSA |
| DRE | Digital Rectal Examination |
| IC-ELISA | ImmunoCapture-based ELISA |

| | |
|---|---|
| NSFC | NanoScale Flow Cytometry |
| NTA | Nanoparticle Tracking Analysis |
| EXOMIX | IC-ELISA/NSFC consensus score |
| PC | Principal Component analysis |
| ROC | Receiver Operating Characteristic |
| AUC | Area Under the Curve |

**Background**

Prostate cancer is one of the most commonly diagnosed male cancer (with 1276106 new cases diagnosed worldwide in 2018) with 358989 deaths in 2018 (https://gco.iarc.fr). Currently the diagnosis of prostate cancer is mainly based on rectal exploration, Prostate-Specific Antigen (PSA) levels in the blood and eco-guided biopsy. Mass screening based on the PSA assay remains a controversial topic in urological clinical practice. In fact, there is currently no universally accepted cut-off value and a high PSA value.

PSA test was first approved by US Food and Drug Administration in late 1980s to monitor men suffering from prostate cancer and after ten years was accepted as screening test. PSA is a serine protease made by prostatic epithelial cells and it can be elevated not only in prostate cancer cells but also in other prostate diseases, such as benign prostatic hyperplasia, prostatic infection and prostatic infarction (https://www.cancer.gov).

Attempts to increase the sensitivity of PSA as a marker have been in vain since a reduction in its specificity has been achieved causing a problem of "overdiagnosis" and "overtreatment" in the last decade (Hoffman, 2011; Etzioni & Feuer, 2008; Schroder et al, 2009; Draisma et al, 2009). PSA test has been exposed and is exposing men to overdiagnosis leading to overtreatment, in turn exposing men unnecessarily to complication and side effects of treatments, including surgery and radiation therapy (https://www.cancer.gov).

PSA values above 4.0 ng per milliliter are considered abnormal, however, cutoff levels can change with age, race and individual fisiological condition. In fact, men without cancer from different ethnic and racial groups have different average PSA concentrations (Hoffman, 2011; Lilja, 1985; Taichman et al, 2007; Etzioni & Feuer, 2008; Schroder et al, 2009), and also weight appears to be associated with PSA concentration. In population-based studies of men without prostate cancer, increasing body mass index (BMI) is associated with a lower mean PSA concentration.

Therefore, in clinical practice, it is necessary to identify new biomarkers able to better detect prostate cancer, while simultaneously reducing the number of unnecessary biopsies and the related stress (Andriole et al, 2009; Fall et al, 2009).

For about two decades, biomedical research underwent a sort of earthquake by the discovery that the cells of practically all the organs and systems of the human body release micro-sized and nano-sized vesicles, also called extracellular vesicles (EVs). Cancer cells release increased amounts of these nano-vesicles also called exosomes, which represent "shuttles" for a variety of molecules, including proteins and nucleic acids (Yáñez-Mó et al, 2015; Fais et al, 2016; van Niel et al, 2018; Turchinovich et al, 2019). After their extracellular release, exosomes circulate throughout the body and are detectable in almost all accessible body fluids, including plasma. Of particular interest, the extracellular vesicles released by human tumor cells of different origins express tumor biomarkers usually used in the follow-up of patients with tumors, such as the MART-1 for melanoma or the CEA for colon cancer. Even prostate cancer cells release extracellular vesicles called "prostasomes", which can be detected in the urine or plasma of patients.

Recent studies have shown that circulating exosomes can be characterized and quantified (Logozzi et al, 2009; Duijvesz et al, 2015; Logozzi et al, 2017;). Furthermore, it appears that high plasma levels of exosomes may represent valuable tumor markers (Cappello et al, 2017).

Therefore, exosomes seem to represent a source of specific biomarkers of potential great impact for clinical practice (Fais et al, 2016, Shah et al, 2018) and as shuttles for therapeutic molecules as well (Fais et al, 2013). Exosomes are secreted physiologically by a variety of cells and their release is dramatically increased in tumor cells by some microenvironmental factors, such

extracellular low pH (Parolini et al, 2009), and independently from their tumor nature (Logozzi et al Cancers 2018).

Our hypothesis was that exosomes might shuttle PSA and that the exosome-associated PSA may help in distinguishing both healthy and BPH from prostate cancer patients. We tested this hypothesis in a pilot study, where we set up a methodological approach for measuring exosomes expressing PSA in the plasma of prostate cancer patients. First we showed that microenvironmental acidity induced a release of exosomes expressing PSA (Exo-PSA) by a prostate cancer cell line (LNCaP). This result was consistent to the plasma levels of Exo-PSA that was significantly higher in prostate cancer patients as compared to patients with benign prostatic hypertrophy and controls. These data were obtained with two different methodologies: immunocapture-based ELISA and nano scales flow cytometry; together with the use of Nanosight Tracking Analysis (NTA) for quality control of the EVs purification obtained by UC of plasma samples (Logozzi et al, 2017).

The aim of this study was to extend the preliminary data to a clinical investigation performed in 240 individuals: patients with prostate cancer (80), benign prostatic hypertrophy (80) and healthy donors (80); this in order to verify the possibility to use the plasmatic levels of Exo-PSA for both screening tests and the clinical follow-up of prostate cancer.

**Rationale and Objectives**

A. Rational for the Study. Current Prostate-Specific Antigen (PSA) test does not provide reliable indications of prostatic pathology. We evaluate measurement of exosome-associated PSA (Exo-PSA) as a novel strategy to discriminate benign from aggressive PCa.

B. Expected results. We expect this study to have significant implications for patient health. If there is an increase of exosome-associated PSA (Exo-PSA) from plasma of prostate cancer patients (PCa), we will obtain a novel diagnostic test to discriminate BPH from PCa, without additional clinical and/or diagnostic examinations for patients or treatment or modification of an ongoing treatment, to avoid unnecessary biopsies.

C. Study Objectives

The objective of this study is quantify plasma levels of Exo-PSA in patients with BPH and PCa in order to identify a cut-off for future population studies and to validate exosomal quantification as a new screening test non-invasive prostate cancer. We consider this study to be of enormous importance for new and profitable developments in the early and non-invasive diagnosis of prostate cancer.

*1. Primary endpoint*

- identification and characterization of plasmatic exosomes expressing PSA in patients and controls
- evaluation of differences among PCa patients, BPH patients and healthy subjects in plasmatic exosomes expressing PSA.

*2. Secondary endpoint*

- Correlation among IC-ELISA, NSFC and serum PSA expression

**Study Design**

*A. Study Population*

1. Patients recruitment

All cases enrolled into the study were consecutively included as out-patients referred to our department of urology on the basis of the inclusion criteria. Patients were correctly informed, accepted to be included in the study and signed an informed consensus prior to each procedure.

2. Patient assignment to treatment group

Eligible cases were divided in 3 groups: Control cases (CTR), benign prostatic hyperplasia cases (BPH) and prostate cancer cases (PCa).

**CTR:** 80 male individuals consecutively referred to our department with the following inclusion criteria: age from 18 to 39 years; no clinical evidence of BPH or PCa (digital rectal examination (DRE) and ultrasonography US); prostate volume less than 30 cc; total PSA level less than 1.4 ng/ml; no familiarity for PCa; no therapies that can influence PSA determination.

**BPH:** 80 male individuals consecutively referred to our department with the following characteristics: age from 45 to 75 years, histologically confirmed diagnosis of BPH; no clinical and pathological evidence of PCa; no therapies that can influence PSA determination (es: 5 alpha reductase inhibitors)

**PCa:** 80 male individuals consecutively referred to our department, aged from 45 to 75 years, with a histologically confirmed diagnosis of prostate adenocarcinoma (prostate biopsy). None of cases were submitted to androgen deprivation therapies or other therapies that can influence PSA determination. All cases were stratified in risk classes (EAU classification) on the basis of total PSA levels, Gleason score and clinical stage

3. Inclusion Criteria

**CTR:** male individuals with the following inclusion criteria: age from 18 to 39 years; no clinical evidence of BPH or PCa (digital rectal examination (DRE) and ultrasonography US); prostate volume less than 30 cc; total PSA level less than 1.4 ng/ml.

**BPH:** male individuals with the following inclusion criteria: age from 45 to 75 years, histologically confirmed diagnosis of BPH;

**PCa:** male individuals aged from 45 to 75 years, with a histologically confirmed diagnosis of prostate adenocarcinoma (prostate biopsy).

4. Exclusion Criteria

**CTR:** familiarity for PCa; therapies that can influence PSA determination, acute prostatic inflammation, prostatitis.

**BPH:** clinical or pathological evidence of PCa (PSA determination, DRE, pathological evidence at biopsy or surgery); therapies that can influence PSA determination (es: 5 alpha reductase inhibitors), acute prostatic inflammation, prostatitis.

**PCa:** androgen deprivation therapies, chemotherapies, new generation hormone therapies or other therapies that can influence PSA determination; acute prostatic inflammation, prostatitis.

*B. Study Interventions*

All eligible patients will be offered informed consent, an accurate medical history will be collected and a blood sample will be taken (1 tube of 5 ml). Once collected, the sample will be labeled by the clinical center with an identification code and sent to the Istituto Superiore di Sanità, to the Laboratory of Dr. Mariantonia Logozzi (no later than 2–4 hours) where researchers will be kept and manipulated anonymously in the testing phase with the code assigned by the clinical center.

This is an experimental observational clinical research study in which no additional and/or administered drug tests and/or modified therapy are performed.

*C. Duration of the patients recruitment*

12 months

*D. Patient Safety*

**- Risks to the Safety of Participants Involved in the Study.** No significant physical risks associated with collecting samples. The risks associated with blood sampling are the same as those for normal laboratory sampling.

*E. Study scheme by site*

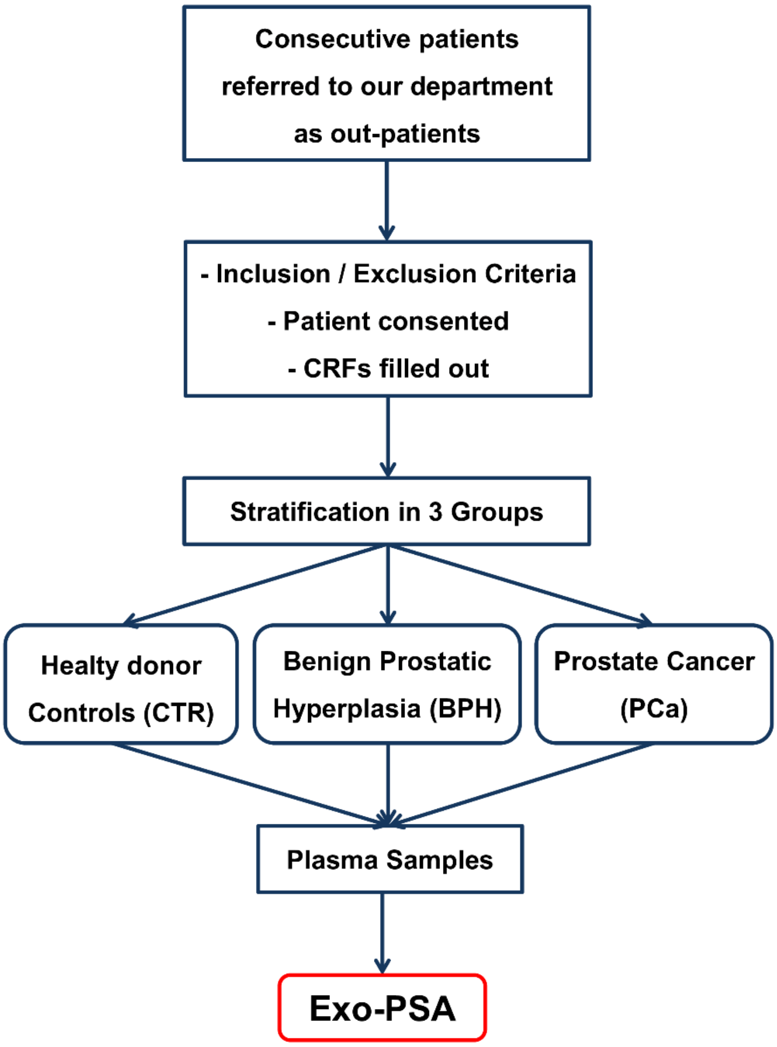


*F. Patients characteristics*

| | CTR | BPH | PCa |
|---|---|---|---|
| Patients, *n*: | 80 | 80 | 80 |
| Age (years): | | | |
| *mean ± SD* | 32.0 ± 4.5 | 67.2 ± 6.5 | 70.9 ± 5.1 |
| *median* | 32 | 69 | 70 |
| *range* | 22–39 | 47–75 | 51–75 |
| Prostate Volume (mL): | | | |
| *mean ± SD* | | 44.2 ± 10.4 | |
| *median* | - | 40.3 | - |
| *range* | | 31–78 | |
| total PSA (ng/ml): | | | |
| *mean ± SD* | | 2.6 ± 1.7 | 8.1 ± 12.8 |
| *median* | | 2.3 | 4.7 |
| *range* | - | 0.3–14.4 | 1.8–100.0 |
| Tumor stage, *n(%)*: | | | |
| *T1-T2 N0 M0* | | | 62 (*77.5%*) |
| *T3 N0 M0* | | | 16 (*20.0%*) |
| *N1* | - | - | 2 (*2.5%*) |
| Gleason score, *n(%)*: | | | |
| *6 (3 + 3)* | | | 33 (*41.3%*) |

| | | | |
|---|---|---|---|
| *7 (3 + 4)* | | | 21 (*26.2%*) |
| *7 (4 + 3)* | | | 15 (*18.8%*) |
| ***8–10*** | - | - | 11 (*13.7%*) |
| **Risk Class, *n(%)*:** | | | |
| ***Low*** | | | 33 (*41.3%*) |
| ***Intermediate*** | | | 18 (*22.5%*) |
| ***High*** | - | - | 29 (*36.2%*) |

*G. Cost*

No additional cost.
This is a non-profit study without funding.

**Study Methods**

Exosomes will be obtained by standard ultracentrifugation (UC) from plasma samples of PCa ($n$ = 80), Benign prostatic hypertrophy (BPH) ($n$ = 80) patients and healthy donor controls (CTR) ($n$ = 80). A quality control of the exosome preparation will be performed by Nanoparticle Tracking Analysis (NTA). Exosome count and characterization will be performed by both immunocapture-based ELISA (IC-ELISA), and Nanoscale Flow-Cytometry (NSFC), for each plasma sample. The statistical analysis will be performed through two main strategies: (i) Computation of pairwise correlation between IC-ELISA, NSFC and serum PSA by means of Pearson correlation coefficient and subsequent generation by Principal Component Analysis of a consensus score summarizing the information shared by the two Exo-PSA methods; (ii) Evaluation of the Receiver Operating Characteristic (ROC) curves in order to evaluate the predictive power of the three different methods for BPH – PCa discrimination.

*A. Preparation of Exosomes from plasma of Patients and Controls*

To obtain exosomes from plasma samples, EDTA-treated blood from patients with prostate cancer (PCa), benign prostatic hyperplasia (BPH) and healthy donors (CTR) will be centrifuged at 400 g for 20 min. Plasma will be then collected and stored at 80 °C until analysis. Upon thawing, 1 ml of plasma samples will be subjected to the same centrifugal procedure as described in Reference (Théry et al., 2018; Caby et al., 2005).

*B. Assays for plasmatic Exosomes characterization and quantification*

1. Nanoparticle tracking analysis

Nanoparticle Tracking Analysis (NTA) from Malvern (NanoSight NS300) will be used for size distribution and concentration measurements of extracellular vesicles samples in liquid suspension from the properties of both light scattering and Brownian motion (Logozzi et al, 2017). The NanoSight NS300 with a 405 nm laser instrument (Malvern Instruments, United Kingdom) will be used to detect exosomes. Five videos of typically 60 s' duration will be taken. Data will be analyzed by NTA 3.0 software (Malvern Instruments) which is optimized to first identify and then track each particle on a frame-by-frame basis. The Brownian motion of each particle is tracked using the Stokes–Einstein equation: $D° = kT/6\pi\eta r$, where D° is the diffusion coefficient, $kT/6\pi\eta r = f0$ is the frictional coefficient of the particle, for the special case of a spherical particle of radius r moving with uniform velocity in a continuous fluid of viscosity $\eta$, k is Boltzmann's constant, and T is the absolute temperature.

2. IC-ELISA for PSA

96 well-plates (Nunc, Milan, Italy) will be coated with 4 μg/ml rabbit polyclonal anti-CD81 antibody (clone PA5–79003, Thermo Fisher Scientific, USA) in 100 μl/well of PBS and incubated overnight at 4 °C. After 3 washes with PBS, 100 μl/well of blocking solution (PBS containing 0.5% BSA) will be added at room temperature for 1 h. Following 3 washes in PBS, exosomes purified

from plasma will be quantified by Bradford assay and then suspended in a final volume of 50 μl and incubated overnight at 37 °C. After 3 washes with PBS, 4 μg/ml of a mouse anti-PSA HRP-conjugated (clone 5A6, Abcam) will be added to each well and incubated for 1 h at 37 °C. After the final 3 washes with PBS, the reaction will be developed with Blue POD for 15 min (Roche Applied Science, Milan), and blocked with 4N $H_2SO_4$ stop solution. Optical densities will be recorded at 450 nm.

3. PSA calibration curve

A PSA calibration curve was previously described (Logozzi et al, 2017). The PSA calibration curve allows to convert the optical densities of each sample into micrograms of Exo-PSA.

Plastic 96 wells strip plates will be coated with 4 μg/ml rabbit polyclonal anti-CD81 antibody (clone PA5–79003, Thermo Fisher Scientific, USA) and incubated overnight at 4 °C. After three washes with PBS, we will perform serial dilutions from 50 μg to 3.1 μg of LNCaP exosome preparation derived from pH 6.5 medium culture and incubate overnight at 37 °C. Samples will be washed three times with PBS, incubated with mouse monoclonal anti-PSA antibody HRP conjugate (clone 5A6, Abcam) at room temperature for 2 h. Subsequently, samples will be washed three times with PBS 1X and incubated with Blue POD substrate (Roche Applied Science, Milan) for 15 min and blocked with 4N $H_2SO_4$ stop solution. Optical densities will be recorded at 450 nm.

4. Flow cytometry analysis of Exosomes

Exosomes purified from plasma will be diluted in PBS in a final volume of 50 μl. Anti-human CD81 allophycocyanin (APC) conjugated (Beckman Coulter; Brea, CA, USA) and anti-human PSA fluorescein (FITC) conjugated (clone 5A6, Abcam) or anti IgG2a APC and IgG1 FITC (Beckman Coulter; Brea, CA, USA) will be added to the exosome preparation at optimal pre-titered concentrations and left for 20 min at RT. 500 μl of PBS will be added to samples before the acquisition on the CytoFLEX flow cytometer (Beckman Coulter, Brea, CA, USA). The cytometer will be calibrated using a mixture of non-fluorescent silica beads and fluorescent (green) latex beads with sizes ranging from 110 nm to 1300 nm. This calibration step enables the determination of the sensitivity and resolution of the flow cytometer (fluorescent latex beads) and the size of extracellular vesicles (silica beads). All samples will be acquired at low flow rate for the same amount of time in order to obtain an estimate of absolute counts of exosomes comparable between various samples. The analysis of the data will be performed with FlowJo software (FlowJo, LLC; Ashland, Oregon, USA) (Logozzi et al, 2017).

*C. Statistical Analysis*

The sample size is calculated, with a 95% confidence interval, with the following statistical formula:

$$n=\frac{1{,}96^2\, P_{att}\, (1-P_{att})}{D^2} = \frac{1{,}96^2 * 0{,}19 * (1-0{,}19)}{0{,}086^2} = 80$$

where the expected prevalence ($P_{att}$) is 19% (Italian tumor register) and the precision (D2) is 8.6%. In this study the sample size will be 80 samples per group (CTR, BPH and PCa).

The goal is to establish a cut-off score: scores above the cut-off are called positives (true positives and false positives), while those below the cut-off are called negatives (true negatives and false negatives).

To decide which is the optimal cut-off score, false positives and false negatives must be taken into account. For this purpose, we will construct a ROC curve (Receiver Operating Characteristics), which is plotted in a diagram of reference of coordinate axes in which the abscissa (horizontal axis) is represented by the values of 1 - specificity and the ordinate (vertical axis) is represented by the values of the sensitivity. The area below this curve (Area Under Curve, AUC) represents an index of test accuracy and has the ability to discern between a healthy and sick population.

Statistical power is the probability of a statistical test to reject the null hypothesis when it is false: the level of significance, i.e. the probability of accepting or rejecting the null hypothesis, has been set at 5%; $p$ <0.05 will be considered as statistically significant, determining the rejection of the null hypothesis.

The discriminant power of the different tests will be assessed by Receiver Operating Characteristic (ROC) curves, allowing to estimate both the average discriminant ability of the test (Area under the curve, AUC) and to select cut-off thresholds maximizing sensitivity (percentage of correctly diagnosed cancer patients) and specificity (percentage of correctly diagnosed hypertrophy patients) (Hanley & McNeil,1982) that, in addition to give a global estimate of the discrimination ability of a diagnostic test, it indicates the most useful threshold to separate the two groups of patients.

Mutual correlations among tests and generation of a consensus score between IC-ELISA and NSFC tests (EXOMIX) will be analyzed by means of Pearson correlation and Principal Component Analysis (Giuliani, 2017).

The statistical analysis of the results obtained will be performed with the SAS System program 9.4 version.

The analysis of the ROC curves will be performed with Sigma Plot 11.2 version.

*D. Experimental scheme*

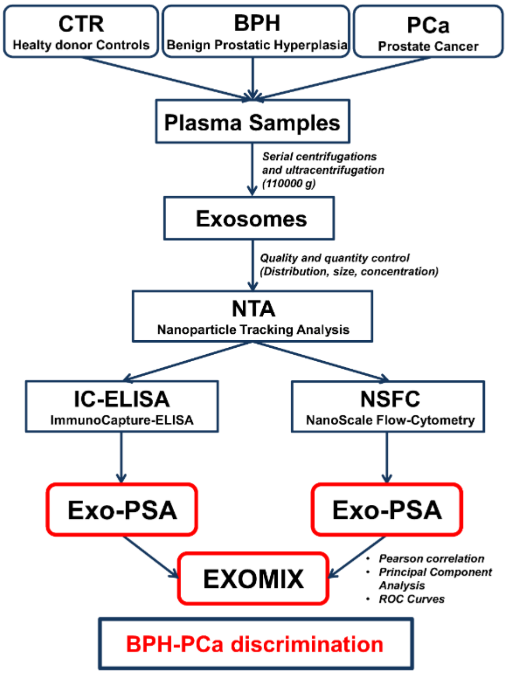


**Statistical methods**

*1. Simple statistics on total data set: MEANS procedure and Coefficient of variation (CV)*

At this first level, we evaluated the coefficient of variation, that is the percentage ratio between standard and average deviation *inside the classes* which provides a synthetic idea of the 'compactness' of the class itself and therefore indirectly of the goodness of the predictive system (obviously combined with the average difference *between the classes*). The lower the coefficient of variation, the greater the efficiency of the discrimination method

Clinical PSA (S-PSA) is unreliable, in PCa and BPH it even has a coefficient of variation greater than 100%, thus making it useless for diagnosis. Log-NSFC (in this helped by the logarithmic transformation that softens the positive outliers) has the most favorable CV, while IC-ELISA prevails (minor CV) on NSFC. However, it should be noted that the softening of the standard

deviation has the 'counterbalance' of the detail decrease (as can be seen from the fact that in Log-NSFC the classes have averages closer to each other).

*2. Correlation on total data set: Pearson correlation coefficient.*

S-PSA is only marginally related to the NSFC value but how this correlation disappears after the latter's logarithmic transformation, suggesting a spurious correlation driven by common outliers. The correlation between NSFC and its logarithm is obvious even if its value is not too high confirms that we did well to do the transform, below we will limit ourselves to this. Finally, the most relevant thing is that the PSA measured on exosomes with IC-ELISA scales well with the NSFC method. This is the most important message of this analysis: the consistency between the two methods of measurement of exosomal PSA and their independence from S-PSA.

*3. Correlation intra-classes.*

The intra-class correlations between S-PSA and NSFC are mild and only apparent. This indicates how the relationship is not necessary (valid also on the small scale) but guided by the variation of the pathological state, which implies that the NSFC method and the IC-ELISA method give a different and potentially complementary view (ie they can be helped in a combined strategy) for discrimination. The link between NSFC and its logarithmic transform is instead higher intra-class than on the complete set but this is only a methodological curiosity linked to the compactness inside the classes and therefore to the approach between the scales (linear and logarithmic) with low variability (within classes).

Returning instead to the complete case it is worth identifying the "latent" variables (main components) of the "PSA" system. The main components look for the "correlated directions of variability" within multivariate systems, in other words the "phenomena underlying a certain data field, hereinafter the result of the factor analysis (method = principal component analysis):

*4. Multidimensional analysis: S-PSA, IC-ELISA, Log-NSFC*

Initial factorial method: Main components

There are only two latent factors (85% of explained variance): the first factor (component) collects the common information of IC-ELISA and log-NSFC, the second (the components are independent by construction), S-PSA. This is the definitive proof that S-PSA does not have anything to see with Exo-PSA (the factorial pattern shows the correlation coefficient between the initial variables and the components). The third component (not shown here) collects the 'noise' of the data field, while the first (Factor1) is Exo-PSA (common part between NSFC and IC-ELISA, therefore the 'optimal' measure of the Exo-PSA regardless of the method used), the second (Factor2) is instead the S-PSA (independent of the first factor).

*5. Construction of an explicit model of calculation of Factor1 (EXOMIX = consensus between the two estimation PSA methods of the Exo-PSA)*

From the above it is interesting to construct an explicit function that allows to estimate the value of Factor1 (which has a mean of zero and unit standard deviation, ie a normalized score) starting from the two IC-ELISA and log-NSFC values (Figure 1), and is what we will do below:

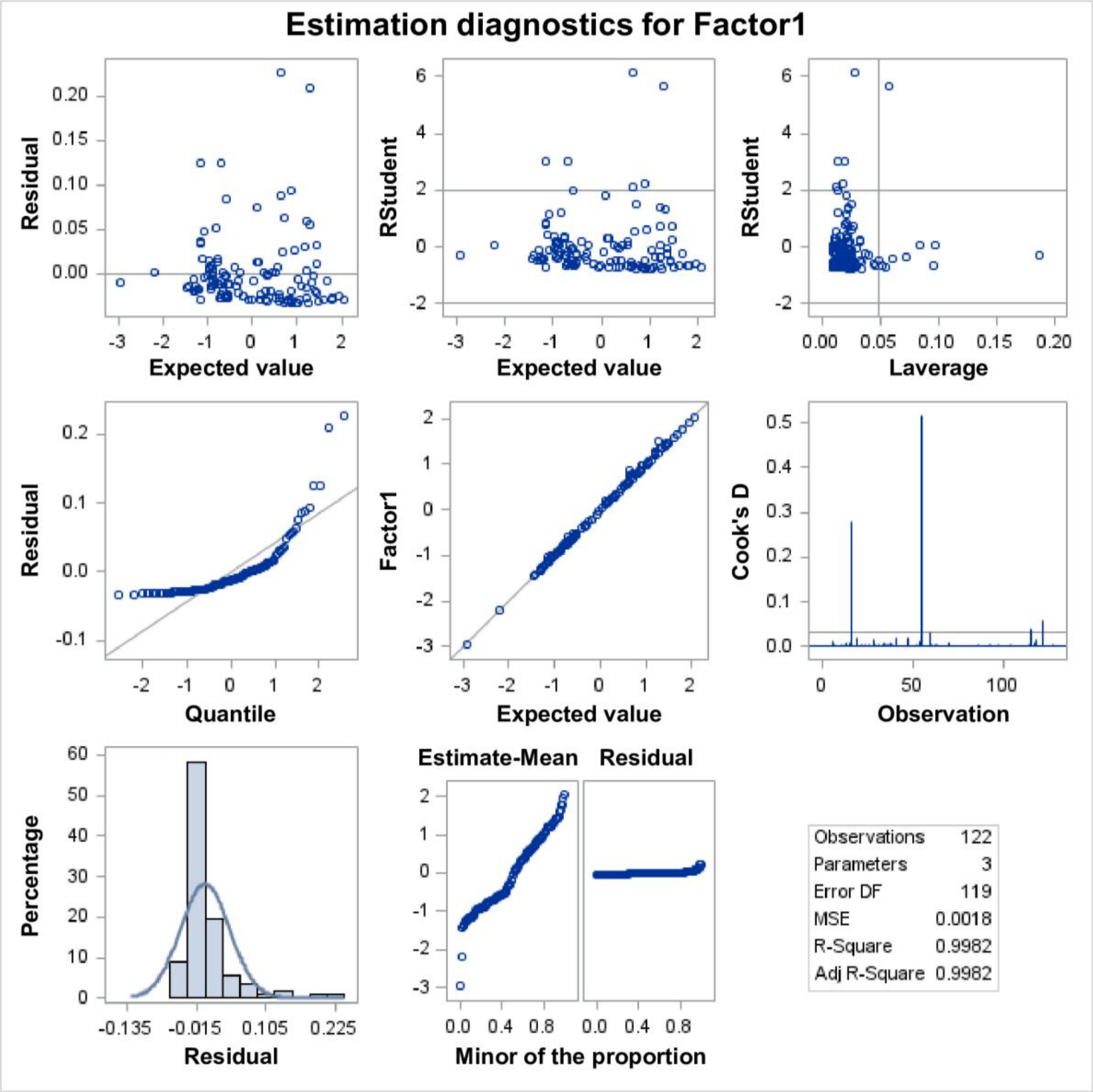


**Figure 1.** Estimation diagnostics for Factor1.

The least squares estimated model:

EXOMIX = −5.28 + 0.022 * IC-ELISA + 0.462 * log-NSFC

It accurately reconstructs the value of the first component (see second graph of the central line) and can be used on any subject whose values of the two methods of analysis are known, replacing the corresponding values of IC-ELISA and Log-NSFC in the equation above.

The (almost) perfect separation between the two classes with EXOMIX > 0 typical of PCa and EXOMIX <0 typical of BPH is already observed at this level.

*6. Discriminating Analysis: IC-ELISA combined with Log-NSFC (EXOMIX).*

The linear discriminant analysis generates the 'linear separator mile' between classes allowing an immediate estimate of the accuracy of a diagnostic criterion.

The EXOMIX composite index system is able to identify 100% of cases in BPH class, and 95.24% in PCa class.

*7. Three-class discriminant analysis (PCa, BPH, CTR) with Log-NSFC.*

The NSFC method allows us to estimate the Exo-PSA also in the controls, this allows to perform a discriminating analysis with the three classes together: consistently with the quadratic

distances between the groups (PCa, BPH and CTR) there is practically no confusion between the classes PCa and CTR while BPH and CTR overlap very much.

*8. Inferential statistics: the TTEST procedure*

The Exo-PSA very well predicts the tumor condition, below the statistical significance: A. Variable NSFC: the two groups (PCa and BPH) are very different in a statistically significant way (averages) with the more variable group PCa (Figure 2).B. Variable S-PSA: Only the relative variability is significantly different between PCa and BPH while the averages are not (Figures 3 and 7).C. Variable IC-ELISA: as in the NSFC case, there is a clear significant difference between the two groups (PCa and BPH) (Figure 4).D. Variable Log-NSFC: A very significant difference between the two groups (PCa and BPH), the logarithm makes the differences in variability disappear within groups (Figure 5).E. Variable EXOMIX (which is the same as Factor1): is the variable with the strongest discrimination (Figures 6 and 8).

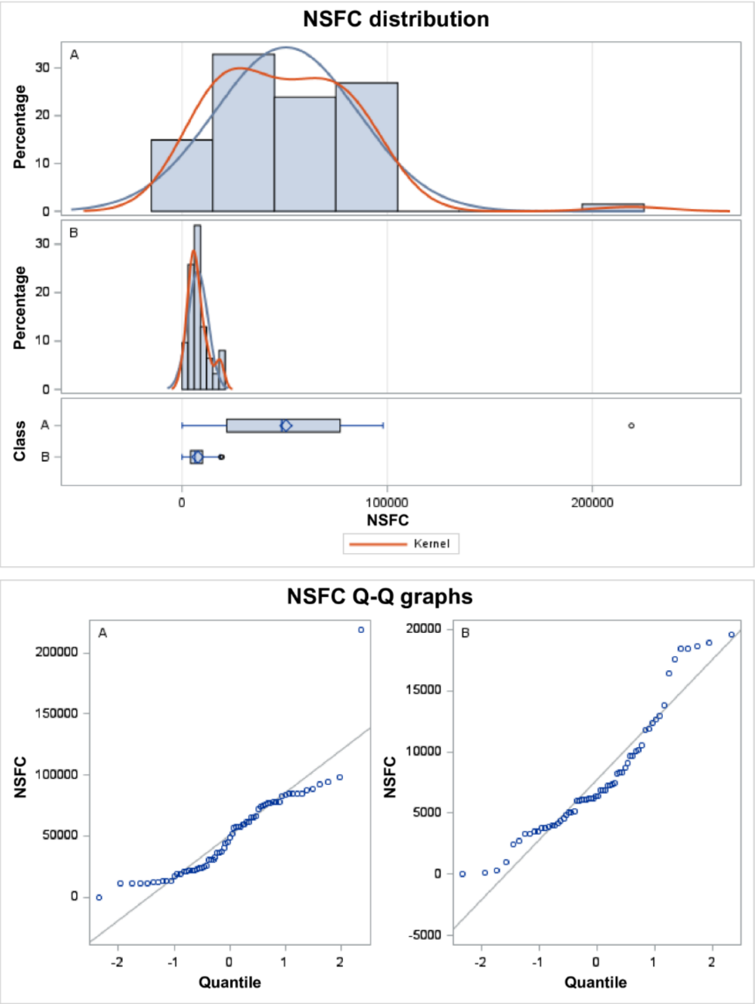


**Figure 2.** NSFC distribution and Q-Q graphs.

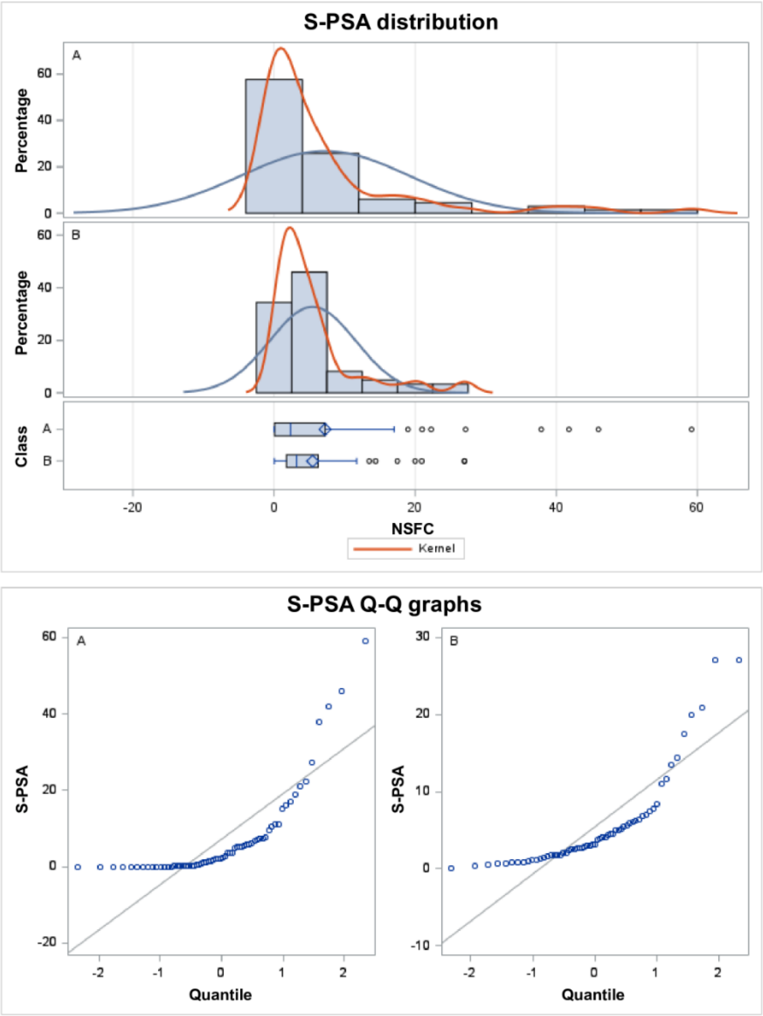


**Figure 3.** S-PSA distribution and Q-Q graphs.

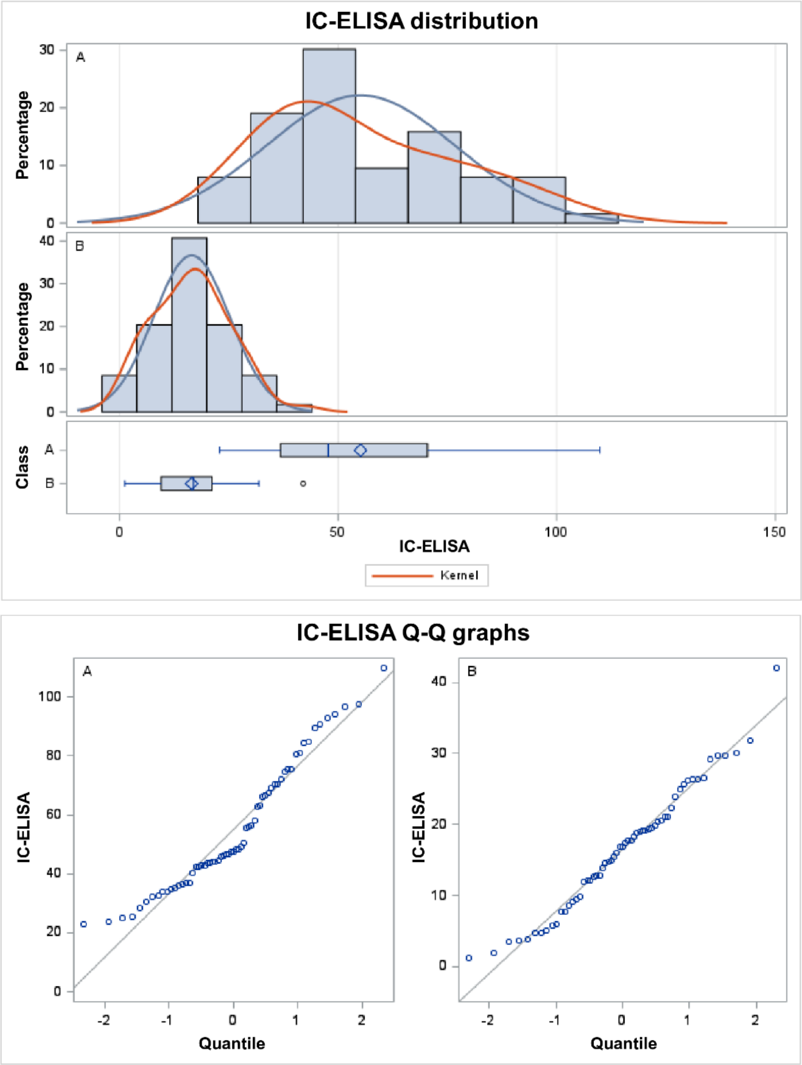


**Figure 4.** IC-ELISA distribution and Q-Q graphs.

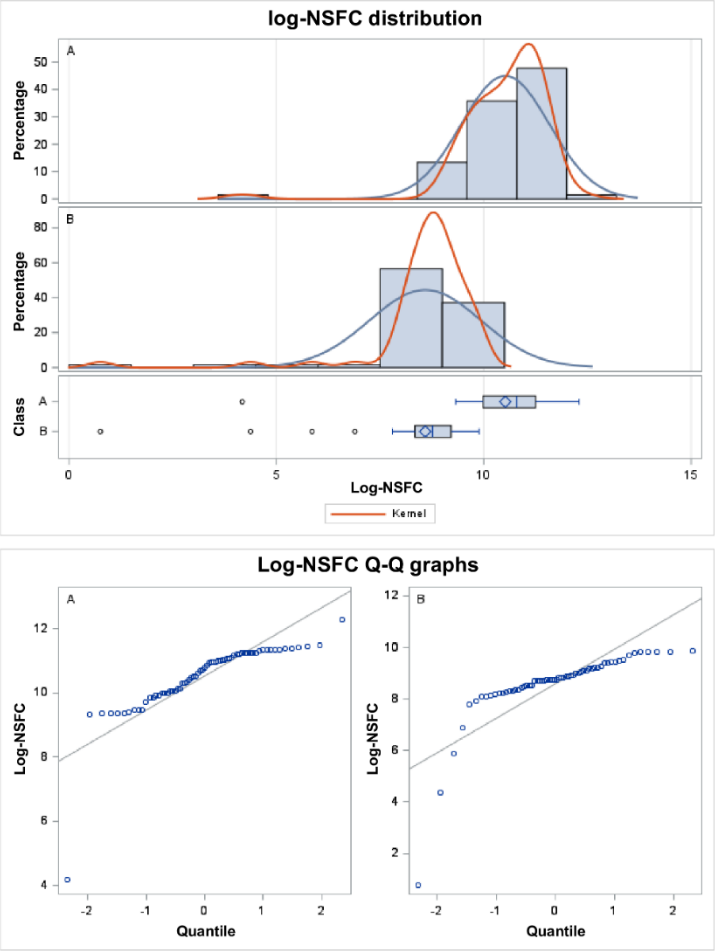


**Figure 5.** Log-NSFC distribution and Q-Q graphs.

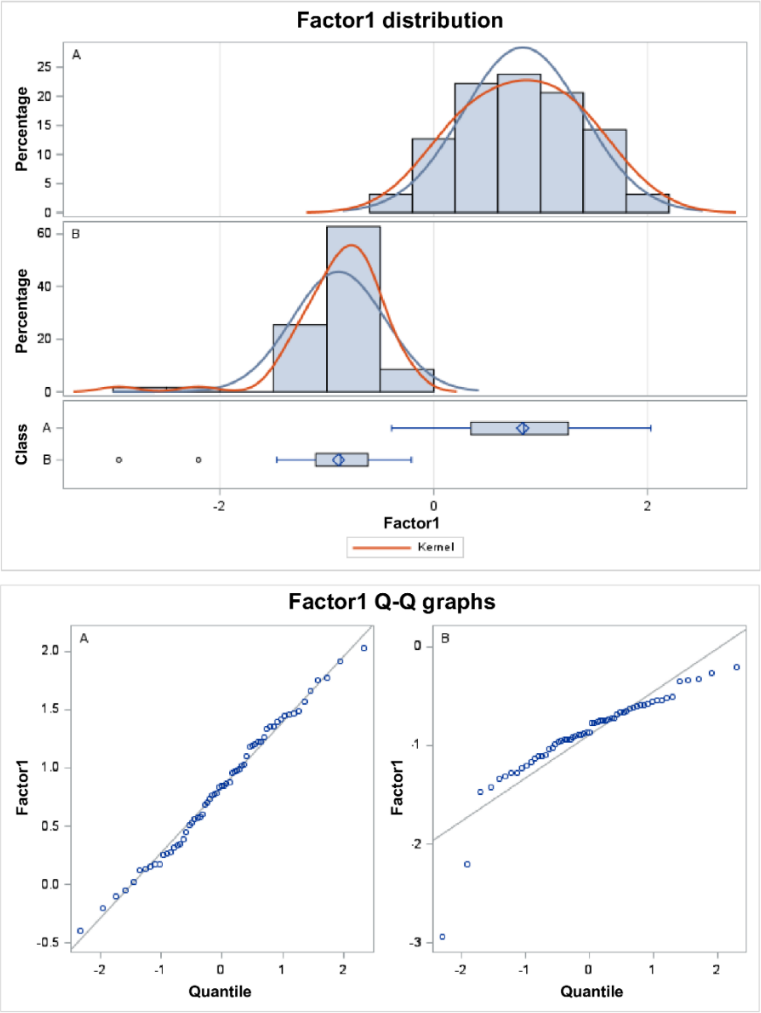


**Figure 6.** Factor1 distribution and Q-Q graphs.

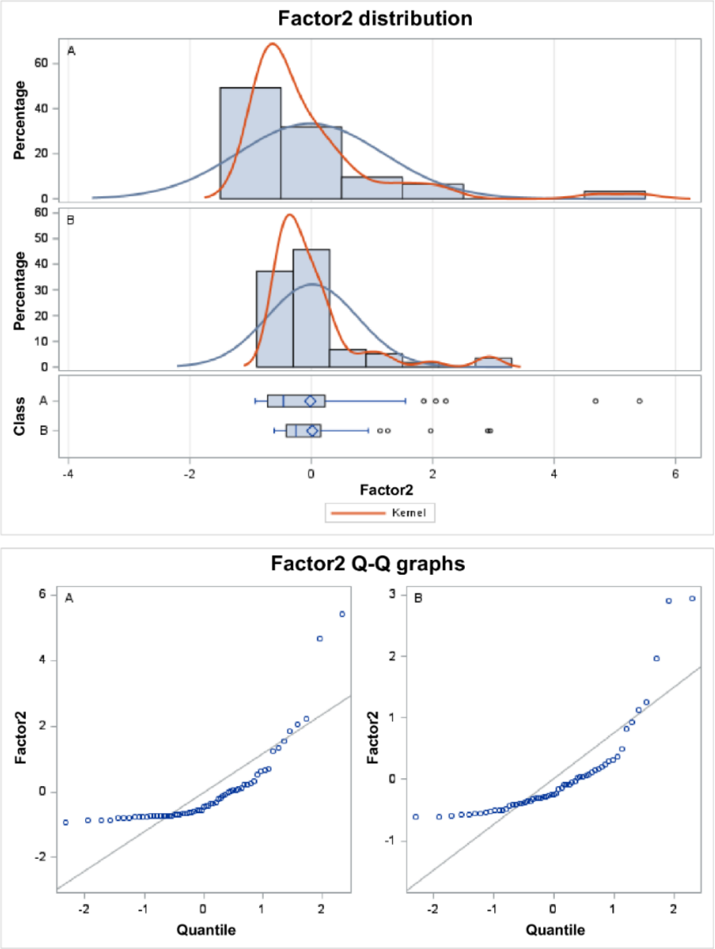


**Figure 7.** Factor2 distribution and Q-Q graphs.

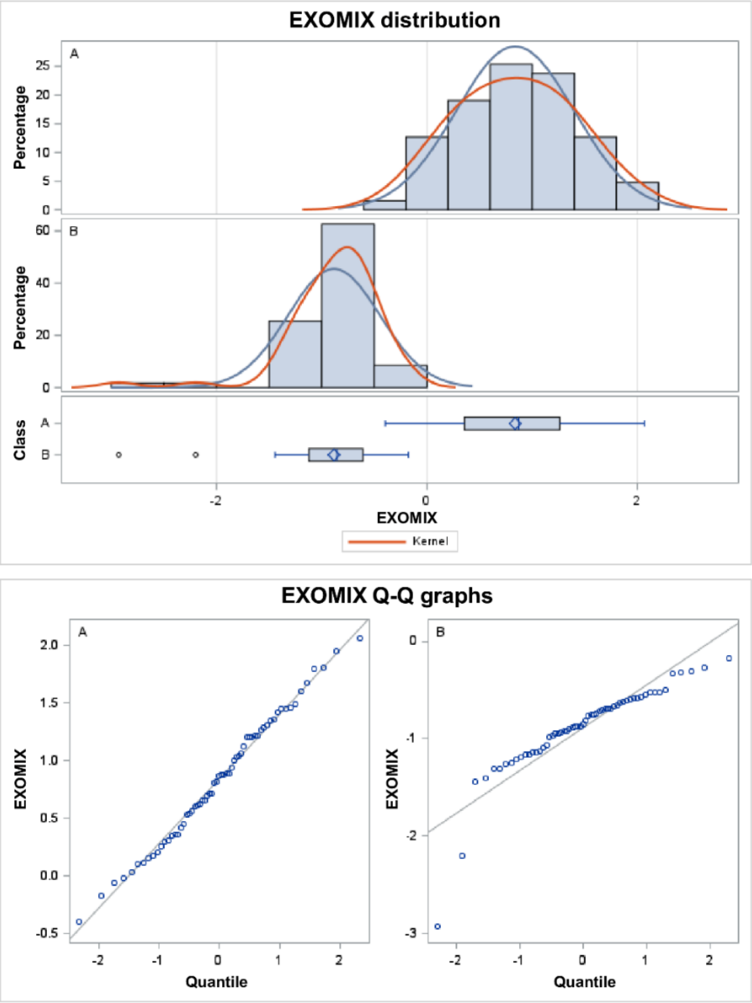


**Figure 8.** EXOMIX distribution and Q-Q graphs.

## ROC Curves

*1. ROC curve IC-ELISA.*

A. ROC Curve IC-ELISA, PCa vs. CTR

**Table A1.**

**a)**

| Sample Size PCa | Sample Size CTR | ROC Curve Area | Standard Error | *p* Value |
|---|---|---|---|---|
| 70 | 70 | **1000** | 0,000 | **< 0.0001** |

**b)**

| Cutoff >. | Sensitivity | 95% CI | Specificity | 95% CI | LR + | LR - | PV + | PV - |
|---|---|---|---|---|---|---|---|---|
| 0.2222 | 1 | 0.9487 to 1.000 | 0.0143 | | 10.145 | 0 | 0.5036 | 1 |
| 0.2672 | 1 | 0.9487 to 1.000 | 0.0286 | | 10.294 | 0 | 0.5072 | 1 |
| 0.3662 | 1 | 0.9487 to 1.000 | 0.0429 | | 10.448 | 0 | 0.5109 | 1 |
| 0.4381 | 1 | 0.9487 to 1.000 | 0.0571 | | 10.606 | 0 | 0.5147 | 1 |
| 0.4741 | 1 | 0.9487 to 1.000 | 0.0714 | | 10.769 | 0 | 0.5185 | 1 |
| 0.5371 | 1 | 0.9487 to 1.000 | 0.0857 | | 10.937 | 0 | 0.5224 | 1 |
| 0.726 | 1 | 0.9487 to 1.000 | 0.1 | | 11.111 | 0 | 0.5263 | 1 |
| 0.897 | 1 | 0.9487 to 1.000 | 0.1143 | | 11.291 | 0 | 0.5303 | 1 |
| 0.924 | 1 | 0.9487 to 1.000 | 0.1286 | | 11.476 | 0 | 0.5344 | 1 |
| 0.9779 | 1 | 0.9487 to 1.000 | 0.1429 | | 11.667 | 0 | 0.5385 | 1 |
| 1.032 | 1 | 0.9487 to 1.000 | 0.1571 | | 11.864 | 0 | 0.5426 | 1 |

| | | | | | | | |
|---|---|---|---|---|---|---|---|
| 1.05 | 1 | 0.9487 to 1.000 | 0.1714 | 12.069 | 0 | 0.5469 | 1 |
| 1.086 | 1 | 0.9487 to 1.000 | 0.1857 | 1.228 | 0 | 0.5512 | 1 |
| 1.149 | 1 | 0.9487 to 1.000 | 0.2 | 1.25 | 0 | 0.5556 | 1 |
| 1.239 | 1 | 0.9487 to 1.000 | 0.2143 | 12.728 | 0 | 0.56 | 1 |
| 1.329 | 1 | 0.9487 to 1.000 | 0.2286 | 12.963 | 0 | 0.5645 | 1 |
| 1.401 | 1 | 0.9487 to 1.000 | 0.2429 | 13.208 | 0 | 0.5691 | 1 |
| 1.5 | 1 | 0.9487 to 1.000 | 0.2571 | 13.461 | 0 | 0.5738 | 1 |
| 1.572 | 1 | 0.9487 to 1.000 | 0.2857 | 1.4 | 0 | 0.5833 | 1 |
| 1.653 | 1 | 0.9487 to 1.000 | 0.3 | 14.286 | 0 | 0.5882 | 1 |
| 1.743 | 1 | 0.9487 to 1.000 | 0.3286 | 14.894 | 0 | 0.5983 | 1 |
| 1.851 | 1 | 0.9487 to 1.000 | 0.3429 | 15.218 | 0 | 0.6035 | 1 |
| 1.968 | 1 | 0.9487 to 1.000 | 0.3571 | 15.555 | 0 | 0.6087 | 1 |
| 2.004 | 1 | 0.9487 to 1.000 | 0.3714 | 15.908 | 0 | 0.614 | 1 |
| 2.112 | 1 | 0.9487 to 1.000 | 0.3857 | 16.279 | 0 | 0.6195 | 1 |
| 2.291 | 1 | 0.9487 to 1.000 | 0.4 | 16.667 | 0 | 0.625 | 1 |
| 2.48 | 1 | 0.9487 to 1.000 | 0.4286 | 17.501 | 0 | 0.6364 | 1 |
| 2.597 | 1 | 0.9487 to 1.000 | 0.4429 | 1.795 | 0 | 0.6422 | 1 |
| 2.723 | 1 | 0.9487 to 1.000 | 0.4571 | 1.842 | 0 | 0.6481 | 1 |
| 2.93 | 1 | 0.9487 to 1.000 | 0.4714 | 18.918 | 0 | 0.6542 | 1 |
| 3.191 | 1 | 0.9487 to 1.000 | 0.5 | 2 | 0 | 0.6667 | 1 |
| 3.38 | 1 | 0.9487 to 1.000 | 0.5143 | 20.589 | 0 | 0.6731 | 1 |
| 3.479 | 1 | 0.9487 to 1.000 | 0.5286 | 21.213 | 0 | 0.6796 | 1 |
| 3.578 | 1 | 0.9487 to 1.000 | 0.5714 | 23.332 | 0 | 0.7 | 1 |
| 3.65 | 1 | 0.9487 to 1.000 | 0.5857 | 24.137 | 0 | 0.7071 | 1 |
| 3.722 | 1 | 0.9487 to 1.000 | 0.6 | 2.5 | 0 | 0.7143 | 1 |
| 3.749 | 1 | 0.9487 to 1.000 | 0.6143 | 25.927 | 0 | 0.7217 | 1 |
| 3.767 | 1 | 0.9487 to 1.000 | 0.6286 | 26.925 | 0 | 0.7292 | 1 |
| 3.803 | 1 | 0.9487 to 1.000 | 0.6429 | 28.003 | 0 | 0.7369 | 1 |
| 4.019 | 1 | 0.9487 to 1.000 | 0.6571 | 29.163 | 0 | 0.7447 | 1 |
| 4.28 | 1 | 0.9487 to 1.000 | 0.6714 | 30.432 | 0 | 0.7527 | 1 |
| 4.406 | 1 | 0.9487 to 1.000 | 0.6857 | 31.817 | 0 | 0.7609 | 1 |
| 4.514 | 1 | 0.9487 to 1.000 | 0.7 | 33.333 | 0 | 0.7692 | 1 |
| 4.586 | 1 | 0.9487 to 1.000 | 0.7143 | 35.002 | 0 | 0.7778 | 1 |
| 4.622 | 1 | 0.9487 to 1.000 | 0.7286 | 36.846 | 0 | 0.7865 | 1 |
| 4.694 | 1 | 0.9487 to 1.000 | 0.7429 | 38.895 | 0 | 0.7955 | 1 |
| 4.802 | 1 | 0.9487 to 1.000 | 0.7714 | 43.745 | 0 | 0.8139 | 1 |
| 5.089 | 1 | 0.9487 to 1.000 | 0.7857 | 46.664 | 0 | 0.8235 | 1 |
| 5.467 | 1 | 0.9487 to 1.000 | 0.8 | 5 | 0 | 0.8333 | 1 |
| 5.737 | 1 | 0.9487 to 1.000 | 0.8143 | 5.385 | 0 | 0.8434 | 1 |
| 6.115 | 1 | 0.9487 to 1.000 | 0.8286 | 58.343 | 0 | 0.8537 | 1 |
| 6.385 | 1 | 0.9487 to 1.000 | 0.8571 | 69.979 | 0 | 0.875 | 1 |
| 6.673 | 1 | 0.9487 to 1.000 | 0.8714 | 7.776 | 0 | 0.8861 | 1 |
| 7.447 | 1 | 0.9487 to 1.000 | 0.8857 | 87.489 | 0 | 0.8974 | 1 |
| 8.157 | 1 | 0.9487 to 1.000 | 0.9 | 10 | 0 | 0.9091 | 1 |
| 8.877 | 1 | 0.9487 to 1.000 | 0.9143 | 116.686 | 0 | 0.9211 | 1 |
| 9.624 | 1 | 0.9487 to 1.000 | 0.9286 | 140.056 | 0 | 0.9334 | 1 |
| 10.09 | 1 | 0.9487 to 1.000 | 0.9429 | 175.131 | 0 | 0.946 | 1 |
| 10.72 | 1 | 0.9487 to 1.000 | 0.9571 | 23.31 | 0 | 0.9589 | 1 |
| 11.14 | 1 | 0.9487 to 1.000 | 0.9714 | 34.965 | 0 | 0.9722 | 1 |
| 11.21 | 1 | 0.9487 to 1.000 | 0.9857 | 699.301 | 0 | 0.9859 | 1 |
| 17.07 | 1 | 0.9487 to 1.000 | 1 | 0.9487 to 1.000 | 0 | 1 | 1 |
| 23.32 | 0.9857 | | 1 | 0.9487 to 1.000 | 0.0143 | 1 | 0.9859 |
| 24.31 | 0.9714 | | 1 | 0.9487 to 1.000 | 0.0286 | 1 | 0.9722 |
| 25.19 | 0.9571 | | 1 | 0.9487 to 1.000 | 0.0429 | 1 | 0.9589 |
| 26.34 | 0.9429 | | 1 | 0.9487 to 1.000 | 0.0571 | 1 | 0.946 |
| 27.64 | 0.9286 | | 1 | 0.9487 to 1.000 | 0.0714 | 1 | 0.9334 |
| 28.34 | 0.9143 | | 1 | 0.9487 to 1.000 | 0.0857 | 1 | 0.9211 |
| 28.88 | 0.9 | | 1 | 0.9487 to 1.000 | 0.1 | 1 | 0.9091 |
| 29.81 | 0.8857 | | 1 | 0.9487 to 1.000 | 0.1143 | 1 | 0.8974 |
| 31.43 | 0.8714 | | 1 | 0.9487 to 1.000 | 0.1286 | 1 | 0.8861 |
| 32.53 | 0.8571 | | 1 | 0.9487 to 1.000 | 0.1429 | 1 | 0.875 |
| 33.25 | 0.8429 | | 1 | 0.9487 to 1.000 | 0.1571 | 1 | 0.8642 |
| 33.88 | 0.8286 | | 1 | 0.9487 to 1.000 | 0.1714 | 1 | 0.8537 |
| 34.45 | 0.8143 | | 1 | 0.9487 to 1.000 | 0.1857 | 1 | 0.8434 |
| 35.19 | 0.8 | | 1 | 0.9487 to 1.000 | 0.2 | 1 | 0.8333 |
| 35.64 | 0.7857 | | 1 | 0.9487 to 1.000 | 0.2143 | 1 | 0.8235 |
| 36.18 | 0.7714 | | 1 | 0.9487 to 1.000 | 0.2286 | 1 | 0.8139 |
| 36.6 | 0.7571 | | 1 | 0.9487 to 1.000 | 0.2429 | 1 | 0.8046 |
| 36.78 | 0.7429 | | 1 | 0.9487 to 1.000 | 0.2571 | 1 | 0.7955 |
| 37.35 | 0.7286 | | 1 | 0.9487 to 1.000 | 0.2714 | 1 | 0.7865 |

| | | | | | | |
|---|---|---|---|---|---|---|
| 39.02 | 0.7143 | 1 | 0.9487 to 1.000 | 0.2857 | 1 | 0.7778 |
| 41.2 | 0.7 | 1 | 0.9487 to 1.000 | 0.3 | 1 | 0.7692 |
| 42.44 | 0.6857 | 1 | 0.9487 to 1.000 | 0.3143 | 1 | 0.7609 |
| 42.66 | 0.6714 | 1 | 0.9487 to 1.000 | 0.3286 | 1 | 0.7527 |
| 42.84 | 0.6571 | 1 | 0.9487 to 1.000 | 0.3429 | 1 | 0.7447 |
| 43.36 | 0.6429 | 1 | 0.9487 to 1.000 | 0.3571 | 1 | 0.7369 |
| 43.88 | 0.6143 | 1 | 0.9487 to 1.000 | 0.3857 | 1 | 0.7217 |
| 44.1 | 0.6 | 1 | 0.9487 to 1.000 | 0.4 | 1 | 0.7143 |
| 44.26 | 0.5857 | 1 | 0.9487 to 1.000 | 0.4143 | 1 | 0.7071 |
| 45.18 | 0.5714 | 1 | 0.9487 to 1.000 | 0.4286 | 1 | 0.7 |
| 46.2 | 0.5571 | 1 | 0.9487 to 1.000 | 0.4429 | 1 | 0.693 |
| 46.46 | 0.5429 | 1 | 0.9487 to 1.000 | 0.4571 | 1 | 0.6863 |
| 46.56 | 0.5286 | 1 | 0.9487 to 1.000 | 0.4714 | 1 | 0.6796 |
| 47.14 | 0.5143 | 1 | 0.9487 to 1.000 | 0.4857 | 1 | 0.6731 |
| 47.68 | 0.5 | 1 | 0.9487 to 1.000 | 0.5 | 1 | 0.6667 |
| 48.02 | 0.4857 | 1 | 0.9487 to 1.000 | 0.5143 | 1 | 0.6604 |
| 48.35 | 0.4714 | 1 | 0.9487 to 1.000 | 0.5286 | 1 | 0.6542 |
| 48.77 | 0.4571 | 1 | 0.9487 to 1.000 | 0.5429 | 1 | 0.6481 |
| 49.87 | 0.4429 | 1 | 0.9487 to 1.000 | 0.5571 | 1 | 0.6422 |
| 53.13 | 0.4286 | 1 | 0.9487 to 1.000 | 0.5714 | 1 | 0.6364 |
| 55.76 | 0.4143 | 1 | 0.9487 to 1.000 | 0.5857 | 1 | 0.6306 |
| 56.06 | 0.4 | 1 | 0.9487 to 1.000 | 0.6 | 1 | 0.625 |
| 57.22 | 0.3857 | 1 | 0.9487 to 1.000 | 0.6143 | 1 | 0.6195 |
| 58.66 | 0.3714 | 1 | 0.9487 to 1.000 | 0.6286 | 1 | 0.614 |
| 61.07 | 0.3571 | 1 | 0.9487 to 1.000 | 0.6429 | 1 | 0.6087 |
| 62.99 | 0.3429 | 1 | 0.9487 to 1.000 | 0.6571 | 1 | 0.6035 |
| 64.22 | 0.3286 | 1 | 0.9487 to 1.000 | 0.6714 | 1 | 0.5983 |
| 65.73 | 0.3143 | 1 | 0.9487 to 1.000 | 0.6857 | 1 | 0.5932 |
| 66.29 | 0.3 | 1 | 0.9487 to 1.000 | 0.7 | 1 | 0.5882 |
| 67 | 0.2857 | 1 | 0.9487 to 1.000 | 0.7143 | 1 | 0.5833 |
| 68.25 | 0.2714 | 1 | 0.9487 to 1.000 | 0.7286 | 1 | 0.5785 |
| 69.69 | 0.2571 | 1 | 0.9487 to 1.000 | 0.7429 | 1 | 0.5738 |
| 70.41 | 0.2429 | 1 | 0.9487 to 1.000 | 0.7571 | 1 | 0.5691 |
| 71.31 | 0.2286 | 1 | 0.9487 to 1.000 | 0.7714 | 1 | 0.5645 |
| 73.51 | 0.2143 | 1 | 0.9487 to 1.000 | 0.7857 | 1 | 0.56 |
| 75.08 | 0.2 | 1 | 0.9487 to 1.000 | 0.8 | 1 | 0.5556 |
| 75.47 | 0.1857 | 1 | 0.9487 to 1.000 | 0.8143 | 1 | 0.5512 |
| 75.92 | 0.1714 | 1 | 0.9487 to 1.000 | 0.8286 | 1 | 0.5469 |
| 78.3 | 0.1571 | 1 | 0.9487 to 1.000 | 0.8429 | 1 | 0.5426 |
| 80.68 | 0.1429 | 1 | 0.9487 to 1.000 | 0.8571 | 1 | 0.5385 |
| 82.72 | 0.1286 | 1 | 0.9487 to 1.000 | 0.8714 | 1 | 0.5344 |
| 84.67 | 0.1143 | 1 | 0.9487 to 1.000 | 0.8857 | 1 | 0.5303 |
| 87.14 | 0.1 | 1 | 0.9487 to 1.000 | 0.9 | 1 | 0.5263 |
| 90.05 | 0.0857 | 1 | 0.9487 to 1.000 | 0.9143 | 1 | 0.5224 |
| 91.74 | 0.0714 | 1 | 0.9487 to 1.000 | 0.9286 | 1 | 0.5185 |
| 93.4 | 0.0571 | 1 | 0.9487 to 1.000 | 0.9429 | 1 | 0.5147 |
| 95.38 | 0.0429 | 1 | 0.9487 to 1.000 | 0.9571 | 1 | 0.5109 |
| 97.07 | 0.0286 | 1 | 0.9487 to 1.000 | 0.9714 | 1 | 0.5072 |
| 103.6 | 0.0143 | 1 | 0.9487 to 1.000 | 0.9857 | 1 | 0.5036 |

B. ROC Curve IC-ELISA, PCa vs. BPH

**Table A2.**

**a)**

| Sample Size PCa | Sample Size BPH | ROC Curve Area | Standard Error | *p* Value |
|---|---|---|---|---|
| 70 | 71 | 0,9821 | 0,007880 | < 0,0001 |

**b)**

| Cutoff > | Sensitivity | 95% CI | Specificity | 95% CI | LR + | LR - | PV + | PV - |
|---|---|---|---|---|---|---|---|---|
| | | | | | | | | |
| 1.599 | 1 | 0.9487 to 1.000 | 0.0141 | | 1.0143 | 0 | 0.5035 | 1 |
| 2.058 | 1 | 0.9487 to 1.000 | 0.0282 | | 1.029 | 0 | 0.5071 | 1 |
| 2.219 | 1 | 0.9487 to 1.000 | 0.0423 | | 1.0441 | 0 | 0.5108 | 1 |
| 2.534 | 1 | 0.9487 to 1.000 | 0.0563 | | 1.0597 | 0 | 0.5145 | 1 |
| 2.894 | 1 | 0.9487 to 1.000 | 0.0704 | | 1.0758 | 0 | 0.5182 | 1 |
| 3.254 | 1 | 0.9487 to 1.000 | 0.0845 | | 1.0923 | 0 | 0.5221 | 1 |
| 3.614 | 1 | 0.9487 to 1.000 | 0.0986 | | 1.1094 | 0 | 0.5259 | 1 |

| | | | | | | | |
|---|---|---|---|---|---|---|---|
| 3.758 | 1 | 0.9487 to 1.000 | 0.1127 | 1.127 | 0 | 0.5299 | 1 |
| 4.262 | 1 | 0.9487 to 1.000 | 0.1268 | 1.1452 | 0 | 0.5338 | 1 |
| 4.757 | 1 | 0.9487 to 1.000 | 0.1408 | 1.1639 | 0 | 0.5379 | 1 |
| 4.883 | 1 | 0.9487 to 1.000 | 0.1549 | 1.1833 | 0 | 0.542 | 1 |
| 5.431 | 1 | 0.9487 to 1.000 | 0.169 | 1.2034 | 0 | 0.5461 | 1 |
| 5.935 | 1 | 0.9487 to 1.000 | 0.1831 | 1.2241 | 0 | 0.5504 | 1 |
| 6.565 | 1 | 0.9487 to 1.000 | 0.1972 | 1.2456 | 0 | 0.5547 | 1 |
| 7.312 | 1 | 0.9487 to 1.000 | 0.2113 | 1.2679 | 0 | 0.5591 | 1 |
| 7.6 | 1 | 0.9487 to 1.000 | 0.2254 | 1.291 | 0 | 0.5635 | 1 |
| 7.743 | 1 | 0.9487 to 1.000 | 0.2394 | 1.3148 | 0 | 0.568 | 1 |
| 8.22 | 1 | 0.9487 to 1.000 | 0.2535 | 1.3396 | 0 | 0.5726 | 1 |
| 8.895 | 1 | 0.9487 to 1.000 | 0.2676 | 1.3654 | 0 | 0.5772 | 1 |
| 9.336 | 1 | 0.9487 to 1.000 | 0.2817 | 1.3922 | 0 | 0.582 | 1 |
| 9.606 | 1 | 0.9487 to 1.000 | 0.2958 | 1.4201 | 0 | 0.5868 | 1 |
| 9.732 | 1 | 0.9487 to 1.000 | 0.3099 | 1.4491 | 0 | 0.5917 | 1 |
| 10.31 | 1 | 0.9487 to 1.000 | 0.3239 | 1.4791 | 0 | 0.5966 | 1 |
| 11.41 | 1 | 0.9487 to 1.000 | 0.338 | 1.5106 | 0 | 0.6017 | 1 |
| 12.02 | 1 | 0.9487 to 1.000 | 0.3521 | 1.5434 | 0 | 0.6068 | 1 |
| 12.09 | 1 | 0.9487 to 1.000 | 0.3662 | 1.5778 | 0 | 0.6121 | 1 |
| 12.39 | 1 | 0.9487 to 1.000 | 0.3803 | 1.6137 | 0 | 0.6174 | 1 |
| 12.74 | 1 | 0.9487 to 1.000 | 0.3944 | 1.6513 | 0 | 0.6228 | 1 |
| 12.83 | 1 | 0.9487 to 1.000 | 0.4085 | 1.6906 | 0 | 0.6283 | 1 |
| 12.86 | 1 | 0.9487 to 1.000 | 0.4225 | 1.7316 | 0 | 0.6339 | 1 |
| 13.42 | 1 | 0.9487 to 1.000 | 0.4366 | 1.7749 | 0 | 0.6396 | 1 |
| 14.28 | 1 | 0.9487 to 1.000 | 0.4507 | 1.8205 | 0 | 0.6455 | 1 |
| 14.68 | 1 | 0.9487 to 1.000 | 0.4648 | 1.8685 | 0 | 0.6514 | 1 |
| 14.82 | 1 | 0.9487 to 1.000 | 0.4789 | 1.919 | 0 | 0.6574 | 1 |
| 15.19 | 1 | 0.9487 to 1.000 | 0.493 | 1.9724 | 0 | 0.6636 | 1 |
| 15.73 | 1 | 0.9487 to 1.000 | 0.507 | 2.0284 | 0 | 0.6698 | 1 |
| 16.43 | 1 | 0.9487 to 1.000 | 0.5211 | 2.0881 | 0 | 0.6762 | 1 |
| 16.91 | 1 | 0.9487 to 1.000 | 0.5352 | 2.1515 | 0 | 0.6827 | 1 |
| 17.14 | 1 | 0.9487 to 1.000 | 0.5493 | 2.2188 | 0 | 0.6893 | 1 |
| 17.41 | 1 | 0.9487 to 1.000 | 0.5634 | 2.2904 | 0 | 0.6961 | 1 |
| 17.59 | 1 | 0.9487 to 1.000 | 0.5775 | 2.3669 | 0 | 0.703 | 1 |
| 17.74 | 1 | 0.9487 to 1.000 | 0.5915 | 2.448 | 0 | 0.71 | 1 |
| 18.06 | 1 | 0.9487 to 1.000 | 0.6056 | 2.5355 | 0 | 0.7172 | 1 |
| 18.6 | 1 | 0.9487 to 1.000 | 0.6197 | 2.6295 | 0 | 0.7245 | 1 |
| 18.94 | 1 | 0.9487 to 1.000 | 0.6338 | 2.7307 | 0 | 0.732 | 1 |
| 19.07 | 1 | 0.9487 to 1.000 | 0.6479 | 2.8401 | 0 | 0.7396 | 1 |
| 19.16 | 1 | 0.9487 to 1.000 | 0.662 | 2.9586 | 0 | 0.7474 | 1 |
| 19.23 | 1 | 0.9487 to 1.000 | 0.6761 | 3.0874 | 0 | 0.7553 | 1 |
| 19.41 | 1 | 0.9487 to 1.000 | 0.6901 | 3.2268 | 0 | 0.7634 | 1 |
| 19.67 | 1 | 0.9487 to 1.000 | 0.7042 | 3.3807 | 0 | 0.7717 | 1 |
| 20.05 | 1 | 0.9487 to 1.000 | 0.7183 | 3.5499 | 0 | 0.7802 | 1 |
| 20.44 | 1 | 0.9487 to 1.000 | 0.7324 | 3.7369 | 0 | 0.7889 | 1 |
| 20.74 | 1 | 0.9487 to 1.000 | 0.7465 | 3.9448 | 0 | 0.7978 | 1 |
| 21.03 | 1 | 0.9487 to 1.000 | 0.7606 | 4.1771 | 0 | 0.8068 | 1 |
| 21.14 | 1 | 0.9487 to 1.000 | 0.7746 | 4.4366 | 0 | 0.8161 | 1 |
| 21.7 | 1 | 0.9487 to 1.000 | 0.7887 | 4.7326 | 0 | 0.8256 | 1 |
| 22.56 | 1 | 0.9487 to 1.000 | 0.8028 | 5.071 | 0 | 0.8353 | 1 |
| 23.32 | 0.9857 | | 0.8028 | 4.9985 | 0.0178 | 0.8333 | 0.9825 |
| 23.82 | 0.9714 | | 0.8028 | 4.926 | 0.0356 | 0.8313 | 0.9656 |
| 24.2 | 0.9714 | | 0.8169 | 5.3053 | 0.035 | 0.8414 | 0.9662 |
| 24.68 | 0.9714 | | 0.831 | 5.7479 | 0.0344 | 0.8518 | 0.9667 |
| 24.95 | 0.9571 | | 0.831 | 5.6633 | 0.0516 | 0.8499 | 0.9509 |
| 25.28 | 0.9571 | | 0.8451 | 6.1788 | 0.0508 | 0.8607 | 0.9517 |
| 25.56 | 0.9429 | | 0.8451 | 6.0872 | 0.0676 | 0.8589 | 0.9367 |
| 25.88 | 0.9429 | | 0.8592 | 6.6967 | 0.0665 | 0.8701 | 0.9377 |
| 26.25 | 0.9429 | | 0.8732 | 7.4361 | 0.0654 | 0.8815 | 0.9386 |
| 26.38 | 0.9429 | | 0.8873 | 8.3665 | 0.0644 | 0.8932 | 0.9395 |
| 26.48 | 0.9429 | | 0.9014 | 9.5629 | 0.0633 | 0.9053 | 0.9404 |
| 26.86 | 0.9429 | | 0.9155 | 11.1586 | 0.0624 | 0.9178 | 0.9413 |
| 27.64 | 0.9286 | | 0.9155 | 10.9893 | 0.078 | 0.9166 | 0.9277 |
| 28.34 | 0.9143 | | 0.9155 | 10.8201 | 0.0936 | 0.9154 | 0.9144 |
| 28.88 | 0.9 | | 0.9155 | 10.6509 | 0.1092 | 0.9142 | 0.9015 |
| 29.2 | 0.8857 | | 0.9155 | 10.4817 | 0.1248 | 0.9129 | 0.889 |
| 29.51 | 0.8857 | | 0.9296 | 12.581 | 0.123 | 0.9264 | 0.8905 |

| | | | | | | | |
|---|---|---|---|---|---|---|---|
| 29.92 | 0.8857 | 0.9577 | | 20.9385 | 0.1193 | 0.9544 | 0.8934 |
| 30.24 | 0.8857 | 0.9718 | | 31.4078 | 0.1176 | 0.9691 | 0.8948 |
| 31.16 | 0.8714 | 0.9718 | | 30.9007 | 0.1323 | 0.9687 | 0.8831 |
| 32.15 | 0.8714 | 0.9859 | | 61.8014 | 0.1304 | 0.9841 | 0.8846 |
| 32.53 | 0.8571 | 0.9859 | | 60.7872 | 0.1449 | 0.9838 | 0.8734 |
| 33.25 | 0.8429 | 0.9859 | | 59.7801 | 0.1593 | 0.9835 | 0.8626 |
| 33.88 | 0.8286 | 0.9859 | | 58.766 | 0.1739 | 0.9833 | 0.8519 |
| 34.45 | 0.8143 | 0.9859 | | 57.7518 | 0.1884 | 0.983 | 0.8415 |
| 35.19 | 0.8 | 0.9859 | | 56.7376 | 0.2029 | 0.9827 | 0.8314 |
| 35.64 | 0.7857 | 0.9859 | | 55.7234 | 0.2174 | 0.9824 | 0.8214 |
| 36.18 | 0.7714 | 0.9859 | | 54.7092 | 0.2319 | 0.982 | 0.8118 |
| 36.6 | 0.7571 | 0.9859 | | 53.695 | 0.2464 | 0.9817 | 0.8023 |
| 36.78 | 0.7429 | 0.9859 | | 52.6879 | 0.2608 | 0.9814 | 0.7932 |
| 37.35 | 0.7286 | 0.9859 | | 51.6738 | 0.2753 | 0.981 | 0.7841 |
| 39.02 | 0.7143 | 0.9859 | | 50.6596 | 0.2898 | 0.9806 | 0.7753 |
| 41.09 | 0.7 | 0.9859 | | 49.6454 | 0.3043 | 0.9803 | 0.7667 |
| 42.14 | 0.7 | 1 | 0.9494 to 1.000 | | 0.3 | 1 | 0.7692 |
| 42.44 | 0.6857 | 1 | 0.9494 to 1.000 | | 0.3143 | 1 | 0.7609 |
| 42.66 | 0.6714 | 1 | 0.9494 to 1.000 | | 0.3286 | 1 | 0.7527 |
| 42.84 | 0.6571 | 1 | 0.9494 to 1.000 | | 0.3429 | 1 | 0.7447 |
| 43.36 | 0.6429 | 1 | 0.9494 to 1.000 | | 0.3571 | 1 | 0.7369 |
| 43.88 | 0.6143 | 1 | 0.9494 to 1.000 | | 0.3857 | 1 | 0.7217 |
| 44.1 | 0.6 | 1 | 0.9494 to 1.000 | | 0.4 | 1 | 0.7143 |
| 44.26 | 0.5857 | 1 | 0.9494 to 1.000 | | 0.4143 | 1 | 0.7071 |
| 45.18 | 0.5714 | 1 | 0.9494 to 1.000 | | 0.4286 | 1 | 0.7 |
| 46.2 | 0.5571 | 1 | 0.9494 to 1.000 | | 0.4429 | 1 | 0.693 |
| 46.46 | 0.5429 | 1 | 0.9494 to 1.000 | | 0.4571 | 1 | 0.6863 |
| 46.56 | 0.5286 | 1 | 0.9494 to 1.000 | | 0.4714 | 1 | 0.6796 |
| 47.14 | 0.5143 | 1 | 0.9494 to 1.000 | | 0.4857 | 1 | 0.6731 |
| 47.68 | 0.5 | 1 | 0.9494 to 1.000 | | 0.5 | 1 | 0.6667 |
| 48.02 | 0.4857 | 1 | 0.9494 to 1.000 | | 0.5143 | 1 | 0.6604 |
| 48.35 | 0.4714 | 1 | 0.9494 to 1.000 | | 0.5286 | 1 | 0.6542 |
| 48.77 | 0.4571 | 1 | 0.9494 to 1.000 | | 0.5429 | 1 | 0.6481 |
| 49.87 | 0.4429 | 1 | 0.9494 to 1.000 | | 0.5571 | 1 | 0.6422 |
| 53.13 | 0.4286 | 1 | 0.9494 to 1.000 | | 0.5714 | 1 | 0.6364 |
| 55.76 | 0.4143 | 1 | 0.9494 to 1.000 | | 0.5857 | 1 | 0.6306 |
| 56.06 | 0.4 | 1 | 0.9494 to 1.000 | | 0.6 | 1 | 0.625 |
| 57.22 | 0.3857 | 1 | 0.9494 to 1.000 | | 0.6143 | 1 | 0.6195 |
| 58.66 | 0.3714 | 1 | 0.9494 to 1.000 | | 0.6286 | 1 | 0.614 |
| 61.07 | 0.3571 | 1 | 0.9494 to 1.000 | | 0.6429 | 1 | 0.6087 |
| 62.99 | 0.3429 | 1 | 0.9494 to 1.000 | | 0.6571 | 1 | 0.6035 |
| 64.22 | 0.3286 | 1 | 0.9494 to 1.000 | | 0.6714 | 1 | 0.5983 |
| 65.73 | 0.3143 | 1 | 0.9494 to 1.000 | | 0.6857 | 1 | 0.5932 |
| 66.29 | 0.3 | 1 | 0.9494 to 1.000 | | 0.7 | 1 | 0.5882 |
| 67 | 0.2857 | 1 | 0.9494 to 1.000 | | 0.7143 | 1 | 0.5833 |
| 68.25 | 0.2714 | 1 | 0.9494 to 1.000 | | 0.7286 | 1 | 0.5785 |
| 69.69 | 0.2571 | 1 | 0.9494 to 1.000 | | 0.7429 | 1 | 0.5738 |
| 70.41 | 0.2429 | 1 | 0.9494 to 1.000 | | 0.7571 | 1 | 0.5691 |
| 71.31 | 0.2286 | 1 | 0.9494 to 1.000 | | 0.7714 | 1 | 0.5645 |
| 73.51 | 0.2143 | 1 | 0.9494 to 1.000 | | 0.7857 | 1 | 0.56 |
| 75.08 | 0.2 | 1 | 0.9494 to 1.000 | | 0.8 | 1 | 0.5556 |
| 75.47 | 0.1857 | 1 | 0.9494 to 1.000 | | 0.8143 | 1 | 0.5512 |
| 75.92 | 0.1714 | 1 | 0.9494 to 1.000 | | 0.8286 | 1 | 0.5469 |
| 78.3 | 0.1571 | 1 | 0.9494 to 1.000 | | 0.8429 | 1 | 0.5426 |
| 80.68 | 0.1429 | 1 | 0.9494 to 1.000 | | 0.8571 | 1 | 0.5385 |
| 82.72 | 0.1286 | 1 | 0.9494 to 1.000 | | 0.8714 | 1 | 0.5344 |
| 84.67 | 0.1143 | 1 | 0.9494 to 1.000 | | 0.8857 | 1 | 0.5303 |
| 87.14 | 0.1 | 1 | 0.9494 to 1.000 | | 0.9 | 1 | 0.5263 |
| 90.05 | 0.0857 | 1 | 0.9494 to 1.000 | | 0.9143 | 1 | 0.5224 |
| 91.74 | 0.0714 | 1 | 0.9494 to 1.000 | | 0.9286 | 1 | 0.5185 |
| 93.4 | 0.0571 | 1 | 0.9494 to 1.000 | | 0.9429 | 1 | 0.5147 |
| 95.38 | 0.0429 | 1 | 0.9494 to 1.000 | | 0.9571 | 1 | 0.5109 |
| 97.07 | 0.0286 | 1 | 0.9494 to 1.000 | | 0.9714 | 1 | 0.5072 |
| 103.6 | 0.0143 | 1 | 0.9494 to 1.000 | | 0.9857 | 1 | 0.5036 |

C. ROC Curve IC-ELISA, BPH vs. CTR

**Table A3.**

**a)**

| Sample Size BPH | Sample Size CTR | ROC Curve Area | Standard Error | *p* Value |
|---|---|---|---|---|
| 71 | 70 | **0,9035** | 0,02520 | **< 0,0001** |

**b)**

| Cutoff > | Sensitivity | 95% CI | Specificity | 95% CI | LR + | LR - | PV + | PV - |
|---|---|---|---|---|---|---|---|---|
| 1.599 | 1 | 0.9487 to 1.000 | 0.0141 | | 1.0143 | 0 | 0.5035 | 1 |
| 2.058 | 1 | 0.9487 to 1.000 | 0.0282 | | 1.029 | 0 | 0.5071 | 1 |
| 2.219 | 1 | 0.9487 to 1.000 | 0.0423 | | 1.0441 | 0 | 0.5108 | 1 |
| 2.534 | 1 | 0.9487 to 1.000 | 0.0563 | | 1.0597 | 0 | 0.5145 | 1 |
| 2.894 | 1 | 0.9487 to 1.000 | 0.0704 | | 1.0758 | 0 | 0.5182 | 1 |
| 3.254 | 1 | 0.9487 to 1.000 | 0.0845 | | 1.0923 | 0 | 0.5221 | 1 |
| 3.614 | 1 | 0.9487 to 1.000 | 0.0986 | | 1.1094 | 0 | 0.5259 | 1 |
| 3.758 | 1 | 0.9487 to 1.000 | 0.1127 | | 1.127 | 0 | 0.5299 | 1 |
| 4.262 | 1 | 0.9487 to 1.000 | 0.1268 | | 1.1452 | 0 | 0.5338 | 1 |
| 4.757 | 1 | 0.9487 to 1.000 | 0.1408 | | 1.1639 | 0 | 0.5379 | 1 |
| 4.883 | 1 | 0.9487 to 1.000 | 0.1549 | | 1.1833 | 0 | 0.542 | 1 |
| 5.431 | 1 | 0.9487 to 1.000 | 0.169 | | 1.2034 | 0 | 0.5461 | 1 |
| 5.935 | 1 | 0.9487 to 1.000 | 0.1831 | | 1.2241 | 0 | 0.5504 | 1 |
| 6.565 | 1 | 0.9487 to 1.000 | 0.1972 | | 1.2456 | 0 | 0.5547 | 1 |
| 7.312 | 1 | 0.9487 to 1.000 | 0.2113 | | 1.2679 | 0 | 0.5591 | 1 |
| 7.6 | 1 | 0.9487 to 1.000 | 0.2254 | | 1.291 | 0 | 0.5635 | 1 |
| 7.743 | 1 | 0.9487 to 1.000 | 0.2394 | | 1.3148 | 0 | 0.568 | 1 |
| 8.22 | 1 | 0.9487 to 1.000 | 0.2535 | | 1.3396 | 0 | 0.5726 | 1 |
| 8.895 | 1 | 0.9487 to 1.000 | 0.2676 | | 1.3654 | 0 | 0.5772 | 1 |
| 9.336 | 1 | 0.9487 to 1.000 | 0.2817 | | 1.3922 | 0 | 0.582 | 1 |
| 9.606 | 1 | 0.9487 to 1.000 | 0.2958 | | 1.4201 | 0 | 0.5868 | 1 |
| 9.732 | 1 | 0.9487 to 1.000 | 0.3099 | | 1.4491 | 0 | 0.5917 | 1 |
| 10.31 | 1 | 0.9487 to 1.000 | 0.3239 | | 1.4791 | 0 | 0.5966 | 1 |
| 11.41 | 1 | 0.9487 to 1.000 | 0.338 | | 1.5106 | 0 | 0.6017 | 1 |
| 12.02 | 1 | 0.9487 to 1.000 | 0.3521 | | 1.5434 | 0 | 0.6068 | 1 |
| 12.09 | 1 | 0.9487 to 1.000 | 0.3662 | | 1.5778 | 0 | 0.6121 | 1 |
| 12.39 | 1 | 0.9487 to 1.000 | 0.3803 | | 1.6137 | 0 | 0.6174 | 1 |
| 12.74 | 1 | 0.9487 to 1.000 | 0.3944 | | 1.6513 | 0 | 0.6228 | 1 |
| 12.83 | 1 | 0.9487 to 1.000 | 0.4085 | | 1.6906 | 0 | 0.6283 | 1 |
| 12.86 | 1 | 0.9487 to 1.000 | 0.4225 | | 1.7316 | 0 | 0.6339 | 1 |
| 13.42 | 1 | 0.9487 to 1.000 | 0.4366 | | 1.7749 | 0 | 0.6396 | 1 |
| 14.28 | 1 | 0.9487 to 1.000 | 0.4507 | | 1.8205 | 0 | 0.6455 | 1 |
| 14.68 | 1 | 0.9487 to 1.000 | 0.4648 | | 1.8685 | 0 | 0.6514 | 1 |
| 14.82 | 1 | 0.9487 to 1.000 | 0.4789 | | 1.919 | 0 | 0.6574 | 1 |
| 15.19 | 1 | 0.9487 to 1.000 | 0.493 | | 1.9724 | 0 | 0.6636 | 1 |
| 15.73 | 1 | 0.9487 to 1.000 | 0.507 | | 2.0284 | 0 | 0.6698 | 1 |
| 16.43 | 1 | 0.9487 to 1.000 | 0.5211 | | 2.0881 | 0 | 0.6762 | 1 |
| 16.91 | 1 | 0.9487 to 1.000 | 0.5352 | | 2.1515 | 0 | 0.6827 | 1 |
| 17.14 | 1 | 0.9487 to 1.000 | 0.5493 | | 2.2188 | 0 | 0.6893 | 1 |
| 17.41 | 1 | 0.9487 to 1.000 | 0.5634 | | 2.2904 | 0 | 0.6961 | 1 |
| 17.59 | 1 | 0.9487 to 1.000 | 0.5775 | | 2.3669 | 0 | 0.703 | 1 |
| 17.74 | 1 | 0.9487 to 1.000 | 0.5915 | | 2.448 | 0 | 0.71 | 1 |
| 18.06 | 1 | 0.9487 to 1.000 | 0.6056 | | 2.5355 | 0 | 0.7172 | 1 |
| 18.6 | 1 | 0.9487 to 1.000 | 0.6197 | | 2.6295 | 0 | 0.7245 | 1 |
| 18.94 | 1 | 0.9487 to 1.000 | 0.6338 | | 2.7307 | 0 | 0.732 | 1 |
| 19.07 | 1 | 0.9487 to 1.000 | 0.6479 | | 2.8401 | 0 | 0.7396 | 1 |
| 19.16 | 1 | 0.9487 to 1.000 | 0.662 | | 2.9586 | 0 | 0.7474 | 1 |
| 19.23 | 1 | 0.9487 to 1.000 | 0.6761 | | 3.0874 | 0 | 0.7553 | 1 |
| 19.41 | 1 | 0.9487 to 1.000 | 0.6901 | | 3.2268 | 0 | 0.7634 | 1 |
| 19.67 | 1 | 0.9487 to 1.000 | 0.7042 | | 3.3807 | 0 | 0.7717 | 1 |
| 20.05 | 1 | 0.9487 to 1.000 | 0.7183 | | 3.5499 | 0 | 0.7802 | 1 |
| 20.44 | 1 | 0.9487 to 1.000 | 0.7324 | | 3.7369 | 0 | 0.7889 | 1 |
| 20.74 | 1 | 0.9487 to 1.000 | 0.7465 | | 3.9448 | 0 | 0.7978 | 1 |
| 21.03 | 1 | 0.9487 to 1.000 | 0.7606 | | 4.1771 | 0 | 0.8068 | 1 |
| 21.14 | 1 | 0.9487 to 1.000 | 0.7746 | | 4.4366 | 0 | 0.8161 | 1 |
| 21.7 | 1 | 0.9487 to 1.000 | 0.7887 | | 4.7326 | 0 | 0.8256 | 1 |
| 22.56 | 1 | 0.9487 to 1.000 | 0.8028 | | 5.071 | 0 | 0.8353 | 1 |
| 23.32 | 0.9857 | | 0.8028 | | 4.9985 | 0.0178 | 0.8333 | 0.9825 |
| 23.82 | 0.9714 | | 0.8028 | | 4.926 | 0.0356 | 0.8313 | 0.9656 |

| | | | | | | | |
|---|---|---|---|---|---|---|---|
| 24.2 | 0.9714 | 0.8169 | | 5.3053 | 0.035 | 0.8414 | 0.9662 |
| 24.68 | 0.9714 | 0.831 | | 5.7479 | 0.0344 | 0.8518 | 0.9667 |
| 24.95 | 0.9571 | 0.831 | | 5.6633 | 0.0516 | 0.8499 | 0.9509 |
| 25.28 | 0.9571 | 0.8451 | | 6.1788 | 0.0508 | 0.8607 | 0.9517 |
| 25.56 | 0.9429 | 0.8451 | | 6.0872 | 0.0676 | 0.8589 | 0.9367 |
| 25.88 | 0.9429 | 0.8592 | | 6.6967 | 0.0665 | 0.8701 | 0.9377 |
| 26.25 | 0.9429 | 0.8732 | | 7.4361 | 0.0654 | 0.8815 | 0.9386 |
| 26.38 | 0.9429 | 0.8873 | | 8.3665 | 0.0644 | 0.8932 | 0.9395 |
| 26.48 | 0.9429 | 0.9014 | | 9.5629 | 0.0633 | 0.9053 | 0.9404 |
| 26.86 | 0.9429 | 0.9155 | | 11.1586 | 0.0624 | 0.9178 | 0.9413 |
| 27.64 | 0.9286 | 0.9155 | | 10.9893 | 0.078 | 0.9166 | 0.9277 |
| 28.34 | 0.9143 | 0.9155 | | 10.8201 | 0.0936 | 0.9154 | 0.9144 |
| 28.88 | 0.9 | 0.9155 | | 10.6509 | 0.1092 | 0.9142 | 0.9015 |
| 29.2 | 0.8857 | 0.9155 | | 10.4817 | 0.1248 | 0.9129 | 0.889 |
| 29.51 | 0.8857 | 0.9296 | | 12.581 | 0.123 | 0.9264 | 0.8905 |
| 29.92 | 0.8857 | 0.9577 | | 20.9385 | 0.1193 | 0.9544 | 0.8934 |
| 30.24 | 0.8857 | 0.9718 | | 31.4078 | 0.1176 | 0.9691 | 0.8948 |
| 31.16 | 0.8714 | 0.9718 | | 30.9007 | 0.1323 | 0.9687 | 0.8831 |
| 32.15 | 0.8714 | 0.9859 | | 61.8014 | 0.1304 | 0.9841 | 0.8846 |
| 32.53 | 0.8571 | 0.9859 | | 60.7872 | 0.1449 | 0.9838 | 0.8734 |
| 33.25 | 0.8429 | 0.9859 | | 59.7801 | 0.1593 | 0.9835 | 0.8626 |
| 33.88 | 0.8286 | 0.9859 | | 58.766 | 0.1739 | 0.9833 | 0.8519 |
| 34.45 | 0.8143 | 0.9859 | | 57.7518 | 0.1884 | 0.983 | 0.8415 |
| 35.19 | 0.8 | 0.9859 | | 56.7376 | 0.2029 | 0.9827 | 0.8314 |
| 35.64 | 0.7857 | 0.9859 | | 55.7234 | 0.2174 | 0.9824 | 0.8214 |
| 36.18 | 0.7714 | 0.9859 | | 54.7092 | 0.2319 | 0.982 | 0.8118 |
| 36.6 | 0.7571 | 0.9859 | | 53.695 | 0.2464 | 0.9817 | 0.8023 |
| 36.78 | 0.7429 | 0.9859 | | 52.6879 | 0.2608 | 0.9814 | 0.7932 |
| 37.35 | 0.7286 | 0.9859 | | 51.6738 | 0.2753 | 0.981 | 0.7841 |
| 39.02 | 0.7143 | 0.9859 | | 50.6596 | 0.2898 | 0.9806 | 0.7753 |
| 41.09 | 0.7 | 0.9859 | | 49.6454 | 0.3043 | 0.9803 | 0.7667 |
| 42.14 | 0.7 | 1 | 0.9494 to 1.000 | | 0.3 | 1 | 0.7692 |
| 42.44 | 0.6857 | 1 | 0.9494 to 1.000 | | 0.3143 | 1 | 0.7609 |
| 42.66 | 0.6714 | 1 | 0.9494 to 1.000 | | 0.3286 | 1 | 0.7527 |
| 42.84 | 0.6571 | 1 | 0.9494 to 1.000 | | 0.3429 | 1 | 0.7447 |
| 43.36 | 0.6429 | 1 | 0.9494 to 1.000 | | 0.3571 | 1 | 0.7369 |
| 43.88 | 0.6143 | 1 | 0.9494 to 1.000 | | 0.3857 | 1 | 0.7217 |
| 44.1 | 0.6 | 1 | 0.9494 to 1.000 | | 0.4 | 1 | 0.7143 |
| 44.26 | 0.5857 | 1 | 0.9494 to 1.000 | | 0.4143 | 1 | 0.7071 |
| 45.18 | 0.5714 | 1 | 0.9494 to 1.000 | | 0.4286 | 1 | 0.7 |
| 46.2 | 0.5571 | 1 | 0.9494 to 1.000 | | 0.4429 | 1 | 0.693 |
| 46.46 | 0.5429 | 1 | 0.9494 to 1.000 | | 0.4571 | 1 | 0.6863 |
| 46.56 | 0.5286 | 1 | 0.9494 to 1.000 | | 0.4714 | 1 | 0.6796 |
| 47.14 | 0.5143 | 1 | 0.9494 to 1.000 | | 0.4857 | 1 | 0.6731 |
| 47.68 | 0.5 | 1 | 0.9494 to 1.000 | | 0.5 | 1 | 0.6667 |
| 48.02 | 0.4857 | 1 | 0.9494 to 1.000 | | 0.5143 | 1 | 0.6604 |
| 48.35 | 0.4714 | 1 | 0.9494 to 1.000 | | 0.5286 | 1 | 0.6542 |
| 48.77 | 0.4571 | 1 | 0.9494 to 1.000 | | 0.5429 | 1 | 0.6481 |
| 49.87 | 0.4429 | 1 | 0.9494 to 1.000 | | 0.5571 | 1 | 0.6422 |
| 53.13 | 0.4286 | 1 | 0.9494 to 1.000 | | 0.5714 | 1 | 0.6364 |
| 55.76 | 0.4143 | 1 | 0.9494 to 1.000 | | 0.5857 | 1 | 0.6306 |
| 56.06 | 0.4 | 1 | 0.9494 to 1.000 | | 0.6 | 1 | 0.625 |
| 57.22 | 0.3857 | 1 | 0.9494 to 1.000 | | 0.6143 | 1 | 0.6195 |
| 58.66 | 0.3714 | 1 | 0.9494 to 1.000 | | 0.6286 | 1 | 0.614 |
| 61.07 | 0.3571 | 1 | 0.9494 to 1.000 | | 0.6429 | 1 | 0.6087 |
| 62.99 | 0.3429 | 1 | 0.9494 to 1.000 | | 0.6571 | 1 | 0.6035 |
| 64.22 | 0.3286 | 1 | 0.9494 to 1.000 | | 0.6714 | 1 | 0.5983 |
| 65.73 | 0.3143 | 1 | 0.9494 to 1.000 | | 0.6857 | 1 | 0.5932 |
| 66.29 | 0.3 | 1 | 0.9494 to 1.000 | | 0.7 | 1 | 0.5882 |
| 67 | 0.2857 | 1 | 0.9494 to 1.000 | | 0.7143 | 1 | 0.5833 |
| 68.25 | 0.2714 | 1 | 0.9494 to 1.000 | | 0.7286 | 1 | 0.5785 |
| 69.69 | 0.2571 | 1 | 0.9494 to 1.000 | | 0.7429 | 1 | 0.5738 |
| 70.41 | 0.2429 | 1 | 0.9494 to 1.000 | | 0.7571 | 1 | 0.5691 |
| 71.31 | 0.2286 | 1 | 0.9494 to 1.000 | | 0.7714 | 1 | 0.5645 |
| 73.51 | 0.2143 | 1 | 0.9494 to 1.000 | | 0.7857 | 1 | 0.56 |
| 75.08 | 0.2 | 1 | 0.9494 to 1.000 | | 0.8 | 1 | 0.5556 |
| 75.47 | 0.1857 | 1 | 0.9494 to 1.000 | | 0.8143 | 1 | 0.5512 |

| | | | | | | |
|---|---|---|---|---|---|---|
| 75.92 | 0.1714 | 1 | 0.9494 to 1.000 | 0.8286 | 1 | 0.5469 |
| 78.3 | 0.1571 | 1 | 0.9494 to 1.000 | 0.8429 | 1 | 0.5426 |
| 80.68 | 0.1429 | 1 | 0.9494 to 1.000 | 0.8571 | 1 | 0.5385 |
| 82.72 | 0.1286 | 1 | 0.9494 to 1.000 | 0.8714 | 1 | 0.5344 |
| 84.67 | 0.1143 | 1 | 0.9494 to 1.000 | 0.8857 | 1 | 0.5303 |
| 87.14 | 0.1 | 1 | 0.9494 to 1.000 | 0.9 | 1 | 0.5263 |
| 90.05 | 0.0857 | 1 | 0.9494 to 1.000 | 0.9143 | 1 | 0.5224 |
| 91.74 | 0.0714 | 1 | 0.9494 to 1.000 | 0.9286 | 1 | 0.5185 |
| 93.4 | 0.0571 | 1 | 0.9494 to 1.000 | 0.9429 | 1 | 0.5147 |
| 95.38 | 0.0429 | 1 | 0.9494 to 1.000 | 0.9571 | 1 | 0.5109 |
| 97.07 | 0.0286 | 1 | 0.9494 to 1.000 | 0.9714 | 1 | 0.5072 |
| 103.6 | 0.0143 | 1 | 0.9494 to 1.000 | 0.9857 | 1 | 0.5036 |

## *2. ROC Curve S-PSA, PCa vs. BPH*

**Table A4.**

**a**)

| **Sample Size PCa** | **Sample Size BPH** | **ROC Curve Area** | **Standard Error** | ***p* Value** |
|---|---|---|---|---|
| 65 | 62 | **0,5816** | 0,05484 | **0,1126** (N.S.) |

**b**)

| **Cutoff >** | **Sensitivity** | **95% CI** | **Specificity** | **95% CI** | **LR +** | **LR -** | **PV +** | **PV -** |
|---|---|---|---|---|---|---|---|---|
| 5.00E−04 | 0.9839 | | 0.0462 | | 1.0315 | 0.3489 | 0.5078 | 0.7414 |
| 1.50E−03 | 0.9839 | | 0.0615 | | 1.0484 | 0.2616 | 0.5118 | 0.7926 |
| 2.50E−03 | 0.9839 | | 0.0769 | | 1.0659 | 0.2093 | 0.5159 | 0.8269 |
| 6.50E−03 | 0.9839 | | 0.1231 | | 1.122 | 0.1308 | 0.5288 | 0.8843 |
| 0.015 | 0.9839 | | 0.1385 | | 1.1421 | 0.1162 | 0.5332 | 0.8959 |
| 0.025 | 0.9839 | | 0.1692 | | 1.1843 | 0.0952 | 0.5422 | 0.9131 |
| 0.04 | 0.9839 | | 0.1846 | | 1.2066 | 0.0872 | 0.5468 | 0.9198 |
| 0.055 | 0.9839 | | 0.2154 | | 1.254 | 0.0747 | 0.5563 | 0.9305 |
| 0.065 | 0.9839 | | 0.2308 | | 1.2791 | 0.0698 | 0.5612 | 0.9348 |
| 0.075 | 0.9839 | | 0.2462 | | 1.3053 | 0.0654 | 0.5662 | 0.9386 |
| 0.085 | 0.9839 | | 0.2769 | | 1.3607 | 0.0581 | 0.5764 | 0.9451 |
| 0.1 | 0.9839 | | 0.3231 | | 1.4535 | 0.0498 | 0.5924 | 0.9525 |
| 0.135 | 0.9839 | | 0.3385 | | 1.4874 | 0.0476 | 0.598 | 0.9546 |
| 0.18 | 0.9839 | | 0.3538 | | 1.5226 | 0.0455 | 0.6036 | 0.9565 |
| 0.26 | 0.9839 | | 0.3692 | | 1.5598 | 0.0436 | 0.6093 | 0.9582 |
| 0.33 | 0.9839 | | 0.3846 | | 1.5988 | 0.0419 | 0.6152 | 0.9598 |
| 0.35 | 0.9677 | | 0.3846 | | 1.5725 | 0.084 | 0.6113 | 0.9225 |
| 0.365 | 0.9516 | | 0.3846 | | 1.5463 | 0.1258 | 0.6073 | 0.8882 |
| 0.395 | 0.9516 | | 0.4 | | 1.586 | 0.121 | 0.6133 | 0.8921 |
| 0.435 | 0.9516 | | 0.4154 | | 1.6278 | 0.1165 | 0.6195 | 0.8956 |
| 0.475 | 0.9355 | | 0.4154 | | 1.6002 | 0.1553 | 0.6154 | 0.8656 |
| 0.525 | 0.9194 | | 0.4308 | | 1.6152 | 0.1871 | 0.6176 | 0.8424 |
| 0.565 | 0.9194 | | 0.4462 | | 1.6602 | 0.1806 | 0.6241 | 0.847 |
| 0.595 | 0.9032 | | 0.4462 | | 1.6309 | 0.2169 | 0.6199 | 0.8217 |
| 0.655 | 0.8871 | | 0.4462 | | 1.6018 | 0.253 | 0.6157 | 0.7981 |
| 0.71 | 0.871 | | 0.4615 | | 1.6175 | 0.2795 | 0.6179 | 0.7815 |
| 0.725 | 0.8548 | | 0.4615 | | 1.5874 | 0.3146 | 0.6135 | 0.7607 |
| 0.75 | 0.8548 | | 0.4769 | | 1.6341 | 0.3045 | 0.6204 | 0.7666 |
| 0.785 | 0.8387 | | 0.4769 | | 1.6033 | 0.3382 | 0.6159 | 0.7473 |
| 0.81 | 0.8226 | | 0.4769 | | 1.5725 | 0.372 | 0.6113 | 0.7289 |
| 0.865 | 0.8065 | | 0.4769 | | 1.5418 | 0.4057 | 0.6066 | 0.7114 |
| 0.945 | 0.8065 | | 0.4923 | | 1.5885 | 0.3931 | 0.6137 | 0.7178 |
| 0.99 | 0.8065 | | 0.5077 | | 1.6382 | 0.3811 | 0.621 | 0.724 |
| 1.03 | 0.8065 | | 0.5231 | | 1.6911 | 0.3699 | 0.6284 | 0.73 |
| 1.085 | 0.7903 | | 0.5231 | | 1.6572 | 0.4009 | 0.6237 | 0.7138 |
| 1.12 | 0.7742 | | 0.5231 | | 1.6234 | 0.4317 | 0.6188 | 0.6985 |
| 1.17 | 0.7581 | | 0.5231 | | 1.5896 | 0.4624 | 0.6138 | 0.6838 |
| 1.225 | 0.7581 | | 0.5385 | | 1.6427 | 0.4492 | 0.6216 | 0.69 |
| 1.33 | 0.7419 | | 0.5385 | | 1.6076 | 0.4793 | 0.6165 | 0.676 |
| 1.46 | 0.7258 | | 0.5385 | | 1.5727 | 0.5092 | 0.6113 | 0.6626 |
| 1.505 | 0.6935 | | 0.5385 | | 1.5027 | 0.5692 | 0.6004 | 0.6373 |
| 1.525 | 0.6935 | | 0.5538 | | 1.5542 | 0.5534 | 0.6085 | 0.6437 |

| | | | | | | | | |
|---|---|---|---|---|---|---|---|---|
| 1.6 | 0.6774 | | 0.5538 | | 1.5182 | 0.5825 | 0.6029 | 0.6319 |
| 1.69 | 0.6613 | | 0.5538 | | 1.4821 | 0.6116 | 0.5971 | 0.6205 |
| 1.73 | 0.6613 | | 0.5692 | | 1.5351 | 0.595 | 0.6055 | 0.6269 |
| 1.77 | 0.6452 | | 0.5692 | | 1.4977 | 0.6233 | 0.5996 | 0.616 |
| 1.84 | 0.629 | | 0.5692 | | 1.4601 | 0.6518 | 0.5935 | 0.6054 |
| 1.985 | 0.6129 | | 0.5692 | | 1.4227 | 0.6801 | 0.5872 | 0.5952 |
| 2.17 | 0.5968 | | 0.5692 | | 1.3853 | 0.7084 | 0.5808 | 0.5854 |
| 2.275 | 0.5806 | | 0.5692 | | 1.3477 | 0.7368 | 0.5741 | 0.5758 |
| 2.325 | 0.5323 | | 0.5692 | | 1.2356 | 0.8217 | 0.5527 | 0.5489 |
| 2.375 | 0.5161 | | 0.5692 | | 1.198 | 0.8501 | 0.545 | 0.5405 |
| 2.45 | 0.4839 | | 0.5692 | | 1.1233 | 0.9067 | 0.529 | 0.5245 |
| 2.555 | 0.4677 | | 0.5846 | | 1.1259 | 0.9105 | 0.5296 | 0.5234 |
| 2.635 | 0.4516 | | 0.5846 | | 1.0871 | 0.9381 | 0.5209 | 0.516 |
| 2.68 | 0.4355 | | 0.5846 | | 1.0484 | 0.9656 | 0.5118 | 0.5087 |
| 2.735 | 0.4194 | | 0.5846 | | 1.0096 | 0.9932 | 0.5024 | 0.5017 |
| 2.87 | 0.4032 | | 0.5846 | | 0.9706 | 1.0209 | 0.4925 | 0.4948 |
| 2.99 | 0.3871 | | 0.5846 | | 0.9319 | 1.0484 | 0.4824 | 0.4882 |
| 3.105 | 0.371 | | 0.5846 | | 0.8931 | 1.0759 | 0.4718 | 0.4817 |
| 3.475 | 0.3387 | | 0.5846 | | 0.8154 | 1.1312 | 0.4491 | 0.4692 |
| 3.83 | 0.3226 | | 0.5846 | | 0.7766 | 1.1587 | 0.4371 | 0.4632 |
| 3.955 | 0.3065 | | 0.5846 | | 0.7378 | 1.1863 | 0.4246 | 0.4574 |
| 4.21 | 0.2903 | | 0.5846 | | 0.6988 | 1.214 | 0.4114 | 0.4517 |
| 4.46 | 0.2742 | | 0.5846 | | 0.6601 | 1.2415 | 0.3976 | 0.4461 |
| 4.585 | 0.2581 | | 0.5846 | | 0.6213 | 1.2691 | 0.3832 | 0.4407 |
| 4.675 | 0.2581 | | 0.6 | | 0.6453 | 1.2365 | 0.3922 | 0.4471 |
| 4.71 | 0.2581 | | 0.6154 | | 0.6711 | 1.2056 | 0.4016 | 0.4534 |
| 4.815 | 0.2581 | | 0.6308 | | 0.6991 | 1.1761 | 0.4114 | 0.4595 |
| 4.925 | 0.2581 | | 0.6462 | | 0.7295 | 1.1481 | 0.4218 | 0.4655 |
| 4.97 | 0.2419 | | 0.6462 | | 0.6837 | 1.1732 | 0.4061 | 0.4602 |
| 5.05 | 0.2258 | | 0.6462 | | 0.6382 | 1.1981 | 0.3896 | 0.4549 |
| 5.175 | 0.2097 | | 0.6462 | | 0.5927 | 1.223 | 0.3721 | 0.4498 |
| 5.235 | 0.2097 | | 0.6615 | | 0.6195 | 1.1947 | 0.3825 | 0.4556 |
| 5.295 | 0.2097 | | 0.6769 | | 0.649 | 1.1675 | 0.3936 | 0.4614 |
| 5.375 | 0.2097 | | 0.6923 | | 0.6815 | 1.1416 | 0.4053 | 0.4669 |
| 5.455 | 0.1774 | | 0.6923 | | 0.5765 | 1.1882 | 0.3657 | 0.457 |
| 5.545 | 0.1613 | | 0.6923 | | 0.5242 | 1.2115 | 0.3439 | 0.4522 |
| 5.64 | 0.1452 | | 0.6923 | | 0.4719 | 1.2347 | 0.3206 | 0.4475 |
| 5.76 | 0.1452 | | 0.7231 | | 0.5244 | 1.1821 | 0.344 | 0.4583 |
| 5.845 | 0.1452 | | 0.7385 | | 0.5553 | 1.1575 | 0.357 | 0.4635 |
| 5.885 | 0.129 | | 0.7385 | | 0.4933 | 1.1794 | 0.3303 | 0.4588 |
| 5.97 | 0.1129 | | 0.7385 | | 0.4317 | 1.2012 | 0.3015 | 0.4543 |
| 6.085 | 0.1129 | | 0.7538 | | 0.4586 | 1.1768 | 0.3144 | 0.4594 |
| 6.185 | 0.0968 | | 0.7538 | | 0.3931 | 1.1982 | 0.2822 | 0.4549 |
| 6.34 | 0.0807 | | 0.7538 | | 0.3276 | 1.2196 | 0.2467 | 0.4505 |
| 6.61 | 0.0807 | | 0.7692 | | 0.3494 | 1.1952 | 0.259 | 0.4555 |
| 6.985 | 0.0645 | | 0.7692 | | 0.2795 | 1.2162 | 0.2185 | 0.4512 |
| 7.225 | 0.0645 | | 0.7846 | | 0.2995 | 1.1923 | 0.2305 | 0.4561 |
| 7.265 | 0.0645 | | 0.8 | | 0.3226 | 1.1694 | 0.2439 | 0.461 |
| 7.465 | 0.0645 | | 0.8154 | | 0.3495 | 1.1473 | 0.259 | 0.4657 |
| 7.68 | 0.0484 | | 0.8154 | | 0.2621 | 1.167 | 0.2077 | 0.4615 |
| 7.72 | 0.0484 | | 0.8308 | | 0.286 | 1.1454 | 0.2224 | 0.4661 |
| 7.775 | 0.0484 | | 0.8462 | | 0.3146 | 1.1246 | 0.2393 | 0.4707 |
| 8.08 | 0.0484 | | 0.8615 | | 0.3494 | 1.1046 | 0.2589 | 0.4752 |
| 9.025 | 0.0323 | | 0.8615 | | 0.2329 | 1.1233 | 0.1889 | 0.471 |
| 10.09 | 0.0323 | | 0.8769 | | 0.2621 | 1.1036 | 0.2076 | 0.4754 |
| 10.51 | 0.0161 | | 0.8769 | | 0.131 | 1.122 | 0.1159 | 0.4713 |
| 10.77 | 0.0161 | | 0.8923 | | 0.1498 | 1.1026 | 0.1303 | 0.4756 |
| 12.7 | 0.0161 | | 0.9077 | | 0.1748 | 1.0839 | 0.1488 | 0.4799 |
| 14.97 | 0 | 0.0 to 0.05776 | 0.9077 | | 0 | 1.1017 | 0 | 0.4758 |
| 15.77 | 0 | 0.0 to 0.05776 | 0.9231 | | 0 | 1.0833 | 0 | 0.48 |
| 16.5 | 0 | 0.0 to 0.05776 | 0.9385 | | 0 | 1.0655 | 0 | 0.4841 |
| 18 | 0 | 0.0 to 0.05776 | 0.9538 | | 0 | 1.0484 | 0 | 0.4882 |
| 32.5 | 0 | 0.0 to 0.05776 | 0.9692 | | 0 | 1.0318 | 0 | 0.4922 |
| 73 | 0 | 0.0 to 0.05776 | 0.9846 | | 0 | 1.0156 | 0 | 0.4961 |

*3. ROC Curve log-NSFC, PCa vs. BPH*

**Table A5.**

**a)**

| Sample Size PCa | Sample Size BPH | ROC Curve Area | Standard Error | *p* Value |
|---|---|---|---|---|
| 67 | 62 | **0,9594** | 0,01758 | **< 0,0001** |

**b)**

| Cutoff > | Sensitivity | Specificity | LR + | LR - | PV + | PV - |
|---|---|---|---|---|---|---|
| 2.47 | 1 | 0.0161 | 1.0164 | 0 | 0.5041 | 1 |
| 4.281 | 0.9851 | 0.0161 | 1.0013 | 0.9237 | 0.5003 | 0.5198 |
| 5.123 | 0.9851 | 0.0323 | 1.0179 | 0.4619 | 0.5044 | 0.6841 |
| 6.38 | 0.9851 | 0.0484 | 1.0352 | 0.3079 | 0.5086 | 0.7646 |
| 7.348 | 0.9851 | 0.0645 | 1.053 | 0.2309 | 0.5129 | 0.8124 |
| 7.854 | 0.9851 | 0.0807 | 1.0715 | 0.1847 | 0.5173 | 0.8441 |
| 8.005 | 0.9851 | 0.0968 | 1.0906 | 0.154 | 0.5217 | 0.8666 |
| 8.105 | 0.9851 | 0.1129 | 1.1105 | 0.132 | 0.5262 | 0.8834 |
| 8.134 | 0.9851 | 0.129 | 1.131 | 0.1155 | 0.5307 | 0.8965 |
| 8.161 | 0.9851 | 0.1452 | 1.1524 | 0.1026 | 0.5354 | 0.9069 |
| 8.196 | 0.9851 | 0.1613 | 1.1746 | 0.0924 | 0.5401 | 0.9154 |
| 8.236 | 0.9851 | 0.1774 | 1.1975 | 0.084 | 0.5449 | 0.9225 |
| 8.252 | 0.9851 | 0.1935 | 1.2215 | 0.077 | 0.5498 | 0.9285 |
| 8.278 | 0.9851 | 0.2097 | 1.2465 | 0.0711 | 0.5549 | 0.9337 |
| 8.318 | 0.9851 | 0.2419 | 1.2994 | 0.0616 | 0.5651 | 0.942 |
| 8.358 | 0.9851 | 0.2581 | 1.3278 | 0.0577 | 0.5704 | 0.9454 |
| 8.404 | 0.9851 | 0.2742 | 1.3573 | 0.0543 | 0.5758 | 0.9485 |
| 8.457 | 0.9851 | 0.2903 | 1.3881 | 0.0513 | 0.5812 | 0.9512 |
| 8.499 | 0.9851 | 0.3065 | 1.4205 | 0.0486 | 0.5869 | 0.9536 |
| 8.521 | 0.9851 | 0.3226 | 1.4542 | 0.0462 | 0.5925 | 0.9559 |
| 8.533 | 0.9851 | 0.3387 | 1.4896 | 0.044 | 0.5983 | 0.9579 |
| 8.62 | 0.9851 | 0.3548 | 1.5268 | 0.042 | 0.6042 | 0.9597 |
| 8.706 | 0.9851 | 0.3871 | 1.6073 | 0.0385 | 0.6165 | 0.9629 |
| 8.714 | 0.9851 | 0.4032 | 1.6506 | 0.037 | 0.6227 | 0.9644 |
| 8.718 | 0.9851 | 0.4194 | 1.6967 | 0.0355 | 0.6292 | 0.9657 |
| 8.726 | 0.9851 | 0.4355 | 1.7451 | 0.0342 | 0.6357 | 0.9669 |
| 8.733 | 0.9851 | 0.4677 | 1.8506 | 0.0319 | 0.6492 | 0.9691 |
| 8.749 | 0.9851 | 0.4839 | 1.9087 | 0.0308 | 0.6562 | 0.9701 |
| 8.797 | 0.9851 | 0.5161 | 2.0358 | 0.0289 | 0.6706 | 0.9719 |
| 8.832 | 0.9851 | 0.5323 | 2.1063 | 0.028 | 0.6781 | 0.9728 |
| 8.835 | 0.9851 | 0.5484 | 2.1814 | 0.0272 | 0.6857 | 0.9735 |
| 8.862 | 0.9851 | 0.5645 | 2.262 | 0.0264 | 0.6934 | 0.9743 |
| 8.891 | 0.9851 | 0.5806 | 2.3488 | 0.0257 | 0.7014 | 0.975 |
| 8.902 | 0.9851 | 0.5968 | 2.4432 | 0.025 | 0.7096 | 0.9756 |
| 8.915 | 0.9851 | 0.6129 | 2.5448 | 0.0243 | 0.7179 | 0.9763 |
| 8.966 | 0.9851 | 0.629 | 2.6553 | 0.0237 | 0.7264 | 0.9769 |
| 9.017 | 0.9851 | 0.6452 | 2.7765 | 0.0231 | 0.7352 | 0.9774 |
| 9.026 | 0.9851 | 0.6613 | 2.9085 | 0.0225 | 0.7441 | 0.978 |
| 9.054 | 0.9851 | 0.6774 | 3.0536 | 0.022 | 0.7533 | 0.9785 |
| 9.095 | 0.9851 | 0.6935 | 3.214 | 0.0215 | 0.7627 | 0.979 |
| 9.145 | 0.9851 | 0.7097 | 3.3934 | 0.021 | 0.7724 | 0.9794 |
| 9.18 | 0.9851 | 0.7258 | 3.5926 | 0.0205 | 0.7823 | 0.9799 |
| 9.198 | 0.9851 | 0.7419 | 3.8167 | 0.0201 | 0.7924 | 0.9803 |
| 9.22 | 0.9851 | 0.7581 | 4.0723 | 0.0197 | 0.8029 | 0.9807 |
| 9.246 | 0.9851 | 0.7742 | 4.3627 | 0.0192 | 0.8135 | 0.9811 |
| 9.295 | 0.9851 | 0.7903 | 4.6977 | 0.0189 | 0.8245 | 0.9815 |
| 9.341 | 0.9701 | 0.7903 | 4.6261 | 0.0378 | 0.8223 | 0.9635 |
| 9.364 | 0.9552 | 0.7903 | 4.5551 | 0.0567 | 0.82 | 0.9464 |
| 9.369 | 0.9403 | 0.7903 | 4.484 | 0.0755 | 0.8177 | 0.9298 |
| 9.372 | 0.9254 | 0.7903 | 4.413 | 0.0944 | 0.8153 | 0.9137 |
| 9.379 | 0.9254 | 0.8065 | 4.7824 | 0.0925 | 0.8271 | 0.9153 |
| 9.384 | 0.9254 | 0.8226 | 5.2165 | 0.0907 | 0.8391 | 0.9169 |
| 9.389 | 0.9104 | 0.8226 | 5.1319 | 0.1089 | 0.8369 | 0.9018 |
| 9.409 | 0.8955 | 0.8226 | 5.0479 | 0.127 | 0.8347 | 0.8873 |
| 9.435 | 0.8955 | 0.8387 | 5.5518 | 0.1246 | 0.8474 | 0.8892 |
| 9.458 | 0.8955 | 0.8548 | 6.1674 | 0.1223 | 0.8605 | 0.8911 |
| 9.503 | 0.8507 | 0.871 | 6.5946 | 0.1714 | 0.8683 | 0.8537 |

| | | | | | | |
|---|---|---|---|---|---|---|
| 9.621 | 0.8507 | 0.8871 | 7.535 | 0.1683 | 0.8828 | 0.8559 |
| 9.718 | 0.8507 | 0.9032 | 8.7882 | 0.1653 | 0.8978 | 0.8581 |
| 9.752 | 0.8358 | 0.9032 | 8.6343 | 0.1818 | 0.8962 | 0.8462 |
| 9.8 | 0.8358 | 0.9194 | 10.3697 | 0.1786 | 0.912 | 0.8485 |
| 9.824 | 0.8358 | 0.9355 | 12.9581 | 0.1755 | 0.9284 | 0.8507 |
| 9.828 | 0.8358 | 0.9516 | 17.2686 | 0.1726 | 0.9453 | 0.8528 |
| 9.841 | 0.8358 | 0.9677 | 25.8762 | 0.1697 | 0.9628 | 0.8549 |
| 9.851 | 0.8358 | 0.9839 | 51.913 | 0.1669 | 0.9811 | 0.857 |
| 9.852 | 0.8209 | 0.9839 | 50.9876 | 0.182 | 0.9808 | 0.846 |
| 9.868 | 0.806 | 0.9839 | 50.0621 | 0.1972 | 0.9804 | 0.8353 |
| 9.909 | 0.806 | 1 | | 0.194 | 1 | 0.8375 |
| 9.957 | 0.7761 | 1 | | 0.2239 | 1 | 0.8171 |
| 9.983 | 0.7612 | 1 | | 0.2388 | 1 | 0.8072 |
| 9.989 | 0.7463 | 1 | | 0.2537 | 1 | 0.7976 |
| 10.02 | 0.7313 | 1 | | 0.2687 | 1 | 0.7882 |
| 10.06 | 0.7164 | 1 | | 0.2836 | 1 | 0.7791 |
| 10.07 | 0.7015 | 1 | | 0.2985 | 1 | 0.7701 |
| 10.1 | 0.6866 | 1 | | 0.3134 | 1 | 0.7614 |
| 10.14 | 0.6716 | 1 | | 0.3284 | 1 | 0.7528 |
| 10.23 | 0.6567 | 1 | | 0.3433 | 1 | 0.7444 |
| 10.32 | 0.6418 | 1 | | 0.3582 | 1 | 0.7363 |
| 10.33 | 0.6269 | 1 | | 0.3731 | 1 | 0.7283 |
| 10.37 | 0.6119 | 1 | | 0.3881 | 1 | 0.7204 |
| 10.45 | 0.597 | 1 | | 0.403 | 1 | 0.7128 |
| 10.5 | 0.5821 | 1 | | 0.4179 | 1 | 0.7053 |
| 10.52 | 0.5672 | 1 | | 0.4328 | 1 | 0.6979 |
| 10.57 | 0.5522 | 1 | | 0.4478 | 1 | 0.6907 |
| 10.65 | 0.5373 | 1 | | 0.4627 | 1 | 0.6837 |
| 10.71 | 0.5224 | 1 | | 0.4776 | 1 | 0.6768 |
| 10.75 | 0.5075 | 1 | | 0.4925 | 1 | 0.67 |
| 10.83 | 0.4925 | 1 | | 0.5075 | 1 | 0.6633 |
| 10.9 | 0.4776 | 1 | | 0.5224 | 1 | 0.6569 |
| 10.95 | 0.4627 | 1 | | 0.5373 | 1 | 0.6505 |
| 10.97 | 0.4478 | 1 | | 0.5522 | 1 | 0.6442 |
| 10.99 | 0.4179 | 1 | | 0.5821 | 1 | 0.6321 |
| 11.02 | 0.3881 | 1 | | 0.6119 | 1 | 0.6204 |
| 11.06 | 0.3582 | 1 | | 0.6418 | 1 | 0.6091 |
| 11.08 | 0.3433 | 1 | | 0.6567 | 1 | 0.6036 |
| 11.1 | 0.3284 | 1 | | 0.6716 | 1 | 0.5982 |
| 11.14 | 0.3134 | 1 | | 0.6866 | 1 | 0.5929 |
| 11.2 | 0.2985 | 1 | | 0.7015 | 1 | 0.5877 |
| 11.22 | 0.2836 | 1 | | 0.7164 | 1 | 0.5826 |
| 11.23 | 0.2687 | 1 | | 0.7313 | 1 | 0.5776 |
| 11.25 | 0.2537 | 1 | | 0.7463 | 1 | 0.5726 |
| 11.26 | 0.2239 | 1 | | 0.7761 | 1 | 0.563 |
| 11.3 | 0.1791 | 1 | | 0.8209 | 1 | 0.5492 |
| 11.33 | 0.1642 | 1 | | 0.8358 | 1 | 0.5447 |
| 11.34 | 0.1493 | 1 | | 0.8507 | 1 | 0.5403 |
| 11.37 | 0.0896 | 1 | | 0.9105 | 1 | 0.5234 |
| 11.39 | 0.0746 | 1 | | 0.9254 | 1 | 0.5194 |
| 11.41 | 0.0597 | 1 | | 0.9403 | 1 | 0.5154 |
| 11.44 | 0.0448 | 1 | | 0.9552 | 1 | 0.5115 |
| 11.47 | 0.0299 | 1 | | 0.9702 | 1 | 0.5076 |
| 11.89 | 0.0149 | 1 | | 0.9851 | 1 | 0.5038 |

## *4. ROC Curve EXOMIX. PCA vs. BPH*

$$\text{EXOMIX} = -5.28 + 0.0222{*}\text{IC-ELISA} + 0.462{*}\text{log-NSFC}$$

**Table A6.**

**a**)

| Sample Size PCa | Sample Size BPH | ROC Curve Area | Standard Error | *p* Value |
|---|---|---|---|---|
| 63 | 59 | **0.9987** | 0.001561 | **< 0.0001** |

**b**)

| Cutoff > | Sensitivity | Specificity | LR + | LR - | PV + | PV - |
|---|---|---|---|---|---|---|
| −2.562 | 1 | 0.017 | 1.0172 | 0 | 0.5043 | 1 |
| −1.821 | 1 | 0.0339 | 1.0351 | 0 | 0.5086 | 1 |
| −1.423 | 1 | 0.0509 | 1.0536 | 0 | 0.513 | 1 |
| −1.358 | 1 | 0.0678 | 1.0727 | 0 | 0.5175 | 1 |
| −1.31 | 1 | 0.0848 | 1.0926 | 0 | 0.5221 | 1 |
| −1.281 | 1 | 0.1017 | 1.1132 | 0 | 0.5268 | 1 |
| −1.253 | 1 | 0.1186 | 1.1346 | 0 | 0.5315 | 1 |
| −1.23 | 1 | 0.1356 | 1.1569 | 0 | 0.5364 | 1 |
| −1.198 | 1 | 0.1525 | 1.1799 | 0 | 0.5413 | 1 |
| −1.175 | 1 | 0.1695 | 1.2041 | 0 | 0.5463 | 1 |
| −1.162 | 1 | 0.1864 | 1.2291 | 0 | 0.5514 | 1 |
| −1.15 | 1 | 0.2034 | 1.2553 | 0 | 0.5566 | 1 |
| −1.137 | 1 | 0.2203 | 1.2825 | 0 | 0.5619 | 1 |
| −1.128 | 1 | 0.2373 | 1.3111 | 0 | 0.5673 | 1 |
| −1.106 | 1 | 0.2542 | 1.3408 | 0 | 0.5728 | 1 |
| −1.081 | 1 | 0.2712 | 1.3721 | 0 | 0.5784 | 1 |
| −1.028 | 1 | 0.2881 | 1.4047 | 0 | 0.5841 | 1 |
| −0.9773 | 1 | 0.3051 | 1.4391 | 0 | 0.59 | 1 |
| −0.9552 | 1 | 0.322 | 1.4749 | 0 | 0.5959 | 1 |
| −0.9411 | 1 | 0.339 | 1.5129 | 0 | 0.602 | 1 |
| −0.9358 | 1 | 0.3559 | 1.5526 | 0 | 0.6082 | 1 |
| −0.9257 | 1 | 0.3729 | 1.5946 | 0 | 0.6146 | 1 |
| −0.9184 | 1 | 0.3898 | 1.6388 | 0 | 0.621 | 1 |
| −0.9048 | 1 | 0.4068 | 1.6858 | 0 | 0.6277 | 1 |
| −0.8902 | 1 | 0.4237 | 1.7352 | 0 | 0.6344 | 1 |
| −0.878 | 1 | 0.4407 | 1.7879 | 0 | 0.6413 | 1 |
| −0.8686 | 1 | 0.4576 | 1.8437 | 0 | 0.6483 | 1 |
| −0.8681 | 1 | 0.4746 | 1.9033 | 0 | 0.6556 | 1 |
| −0.8605 | 1 | 0.4915 | 1.9666 | 0 | 0.6629 | 1 |
| −0.8342 | 1 | 0.5085 | 2.0346 | 0 | 0.6705 | 1 |
| −0.7898 | 1 | 0.5254 | 2.107 | 0 | 0.6782 | 1 |
| −0.76 | 1 | 0.5424 | 2.1853 | 0 | 0.6861 | 1 |
| −0.7528 | 1 | 0.5593 | 2.2691 | 0 | 0.6941 | 1 |
| −0.7467 | 1 | 0.5763 | 2.3602 | 0 | 0.7024 | 1 |
| −0.7314 | 1 | 0.5932 | 2.4582 | 0 | 0.7108 | 1 |
| −0.712 | 1 | 0.6102 | 2.5654 | 0 | 0.7195 | 1 |
| −0.7018 | 1 | 0.6271 | 2.6817 | 0 | 0.7284 | 1 |
| −0.6984 | 1 | 0.6441 | 2.8098 | 0 | 0.7375 | 1 |
| −0.6966 | 1 | 0.661 | 2.9499 | 0 | 0.7468 | 1 |
| −0.6844 | 1 | 0.678 | 3.1056 | 0 | 0.7564 | 1 |
| −0.6648 | 1 | 0.6949 | 3.2776 | 0 | 0.7662 | 1 |
| −0.6452 | 1 | 0.7119 | 3.471 | 0 | 0.7763 | 1 |
| −0.6249 | 1 | 0.7288 | 3.6873 | 0 | 0.7867 | 1 |
| −0.6112 | 1 | 0.7458 | 3.9339 | 0 | 0.7973 | 1 |
| −0.5993 | 1 | 0.7627 | 4.2141 | 0 | 0.8082 | 1 |
| −0.5888 | 1 | 0.7797 | 4.5393 | 0 | 0.8195 | 1 |
| −0.5852 | 1 | 0.7966 | 4.9164 | 0 | 0.831 | 1 |
| −0.5774 | 1 | 0.8136 | 5.3648 | 0 | 0.8429 | 1 |
| −0.5579 | 1 | 0.8305 | 5.8997 | 0 | 0.8551 | 1 |
| −0.5372 | 1 | 0.8475 | 6.5574 | 0 | 0.8677 | 1 |
| −0.5254 | 1 | 0.8644 | 7.3746 | 0 | 0.8806 | 1 |
| −0.5198 | 1 | 0.8814 | 8.4317 | 0 | 0.894 | 1 |
| −0.5096 | 1 | 0.8983 | 9.8328 | 0 | 0.9077 | 1 |
| −0.4484 | 1 | 0.9153 | 11.8064 | 0 | 0.9219 | 1 |
| −0.3651 | 0.9841 | 0.9153 | 11.6187 | 0.0174 | 0.9208 | 0.9829 |
| −0.3263 | 0.9841 | 0.9322 | 14.5147 | 0.0171 | 0.9355 | 0.9832 |
| −0.3123 | 0.9841 | 0.9492 | 19.372 | 0.0168 | 0.9509 | 0.9835 |
| −0.2875 | 0.9841 | 0.9661 | 29.0295 | 0.0165 | 0.9667 | 0.9838 |
| −0.2224 | 0.9841 | 0.9831 | 58.2308 | 0.0162 | 0.9831 | 0.9841 |
| −0.1753 | 0.9841 | 1 | | 0.0159 | 1 | 0.9843 |
| −0.118 | 0.9683 | 1 | | 0.0317 | 1 | 0.9693 |
| −0.0398 | 0.9524 | 1 | | 0.0476 | 1 | 0.9546 |
| 8.21E−03 | 0.9365 | 1 | | 0.0635 | 1 | 0.9403 |
| 0.0675 | 0.9206 | 1 | | 0.0794 | 1 | 0.9264 |
| 0.1071 | 0.9048 | 1 | | 0.0952 | 1 | 0.9131 |
| 0.1326 | 0.8889 | 1 | | 0.1111 | 1 | 0.9 |

| | | | | | |
|---|---|---|---|---|---|
| 0.162 | 0.873 | 1 | 0.127 | 1 | 0.8873 |
| 0.1869 | 0.8571 | 1 | 0.1429 | 1 | 0.875 |
| 0.229 | 0.8413 | 1 | 0.1587 | 1 | 0.863 |
| 0.277 | 0.8254 | 1 | 0.1746 | 1 | 0.8514 |
| 0.3021 | 0.8095 | 1 | 0.1905 | 1 | 0.84 |
| 0.325 | 0.7937 | 1 | 0.2063 | 1 | 0.829 |
| 0.3492 | 0.7778 | 1 | 0.2222 | 1 | 0.8182 |
| 0.3552 | 0.7619 | 1 | 0.2381 | 1 | 0.8077 |
| 0.3862 | 0.746 | 1 | 0.254 | 1 | 0.7974 |
| 0.4333 | 0.7302 | 1 | 0.2698 | 1 | 0.7875 |
| 0.4907 | 0.7143 | 1 | 0.2857 | 1 | 0.7778 |
| 0.539 | 0.6984 | 1 | 0.3016 | 1 | 0.7683 |
| 0.5523 | 0.6825 | 1 | 0.3175 | 1 | 0.759 |
| 0.5808 | 0.6667 | 1 | 0.3333 | 1 | 0.75 |
| 0.6105 | 0.6508 | 1 | 0.3492 | 1 | 0.7412 |
| 0.6233 | 0.6349 | 1 | 0.3651 | 1 | 0.7325 |
| 0.6395 | 0.619 | 1 | 0.381 | 1 | 0.7241 |
| 0.654 | 0.6032 | 1 | 0.3968 | 1 | 0.7159 |
| 0.6741 | 0.5873 | 1 | 0.4127 | 1 | 0.7079 |
| 0.7024 | 0.5714 | 1 | 0.4286 | 1 | 0.7 |
| 0.7173 | 0.5556 | 1 | 0.4444 | 1 | 0.6923 |
| 0.7622 | 0.5397 | 1 | 0.4603 | 1 | 0.6848 |
| 0.812 | 0.5238 | 1 | 0.4762 | 1 | 0.6774 |
| 0.846 | 0.5079 | 1 | 0.4921 | 1 | 0.6702 |
| 0.8775 | 0.4921 | 1 | 0.5079 | 1 | 0.6632 |
| 0.8832 | 0.4762 | 1 | 0.5238 | 1 | 0.6563 |
| 0.8877 | 0.4603 | 1 | 0.5397 | 1 | 0.6495 |
| 0.8928 | 0.4444 | 1 | 0.5556 | 1 | 0.6428 |
| 0.919 | 0.4286 | 1 | 0.5714 | 1 | 0.6364 |
| 0.9725 | 0.4127 | 1 | 0.5873 | 1 | 0.63 |
| 1.016 | 0.3968 | 1 | 0.6032 | 1 | 0.6238 |
| 1.038 | 0.381 | 1 | 0.619 | 1 | 0.6177 |
| 1.053 | 0.3651 | 1 | 0.6349 | 1 | 0.6117 |
| 1.091 | 0.3492 | 1 | 0.6508 | 1 | 0.6058 |
| 1.161 | 0.3333 | 1 | 0.6667 | 1 | 0.6 |
| 1.204 | 0.3175 | 1 | 0.6825 | 1 | 0.5944 |
| 1.207 | 0.3016 | 1 | 0.6984 | 1 | 0.5888 |
| 1.211 | 0.2857 | 1 | 0.7143 | 1 | 0.5833 |
| 1.214 | 0.2698 | 1 | 0.7302 | 1 | 0.578 |
| 1.238 | 0.254 | 1 | 0.746 | 1 | 0.5727 |
| 1.274 | 0.2381 | 1 | 0.7619 | 1 | 0.5676 |
| 1.295 | 0.2222 | 1 | 0.7778 | 1 | 0.5625 |
| 1.328 | 0.2063 | 1 | 0.7937 | 1 | 0.5575 |
| 1.357 | 0.1905 | 1 | 0.8095 | 1 | 0.5526 |
| 1.392 | 0.1746 | 1 | 0.8254 | 1 | 0.5478 |
| 1.433 | 0.1587 | 1 | 0.8413 | 1 | 0.5431 |
| 1.447 | 0.1429 | 1 | 0.8571 | 1 | 0.5385 |
| 1.452 | 0.127 | 1 | 0.873 | 1 | 0.5339 |
| 1.472 | 0.1111 | 1 | 0.8889 | 1 | 0.5294 |
| 1.546 | 0.0952 | 1 | 0.9048 | 1 | 0.525 |
| 1.641 | 0.0794 | 1 | 0.9206 | 1 | 0.5207 |
| 1.735 | 0.0635 | 1 | 0.9365 | 1 | 0.5164 |
| 1.801 | 0.0476 | 1 | 0.9524 | 1 | 0.5122 |
| 1.879 | 0.0318 | 1 | 0.9683 | 1 | 0.5081 |
| 2.008 | 0.0159 | 1 | 0.9841 | 1 | 0.504 |

## References

1. Andriole, G.L.; Crawford, E.D.; Grubb, R.L. 3rd. Mortality results from a randomized prostate-cancer screening trial. *N. Engl. J. Med.* **2009**, *360*, 1310–1319
2. Caby, M.P.; Lankar, D.; Vincendeau-Scherrer, C.; Raposo, G.; Bonnerot, C. Exosomal-like vesicles are present in human blood plasma. *Int. Immunol.* **2005**, *17*, 879–887
3. Cappello, F.; Logozzi, M.; Campanella, C.; Bavisotto, C.C.; Marcilla, A.; Properzi, F.; Fais, S. Reprint of "EXOSOME LEVELS IN HUMAN BODY FLUIDS: A TUMOR MARKER BY THEMSELVES?" *Eur. J. Pharm. Sci.* **2017**, *15*, 64–69.

4. Draisma, G.; Etzioni, R.; Tsodikov, A.; Mariotto, A.; Wever, E.; Gulati, R.; Feuer, E.; de Koning, H. Lead time and overdiagnosis in prostate specific antigen screening: importance of methods and context. *J Natl Cancer Inst.* **2009**, *101*, 374–383.
5. Duijvesz, D.; Versluis, C.Y.; van der Fels, C.A.; Vredenbregt-van den Berg, M.S.; Leivo, J.; Peltola, M.T.; Bangma, C.H.; Pettersson, K.S.; Jenster, G. Immuno-based detection of extracellular vesicles in urine as diagnostic marker for prostate cancer. *Int. J. Cancer* **2015**, *15*, 2869–78.
6. Etzioni, R. & Feuer, E. Studies of prostate cancer mortality: caution advised. *Lancet Oncol.* **2008**, *9*, 407–409.
7. Fais, S.; Logozzi, M.; Lugini, L.; Federici, C.; Azzarito, T.; Zarovni, N.; Chiesi, A. Exosomes: the ideal nanovectors for biodelivery. *Biol. Chem.* **2013**, *394*, 1–15.
8. Fais, S.; O'Driscoll, L.; Borras, F.E.; Buzas, E.; Camussi, G.; Cappello, F.; Carvalho, J.; Cordeiro da Silva, A.; Del Portillo, H.; El Andaloussi, S.; et al. Evidence-Based Clinical Use of Nanoscale Extracellular Vesicles in Nanomedicine. *ACS Nano.* **2016**, *10*, 3886–99.
9. Fall, K.; Fang, F.; Mucci, L.A.; Ye, W.; Andrén, O.; Johansson, J.E.; Andersson, S.O.; Sparén, P.; Klein, G.; Stampfer, M.; et al. Immediate risk for cardiovascular events and suicide following a prostate cancer diagnosis: prospective cohort study. *PLoS Med.* 2009, 6, e1000197, doi:10.1371/journal.pmed.1000197.
10. Giuliani, A. The application of principal component analysis to drug discovery and biomedical data. *Drug Discov. Today* **2017**, *22*, 1069–76.
11. Hanley, J.A.; McNeil, B.J. The meaning and use of the area under a receiver operating characteristic (ROC) curve. *Radiology* **1982**, *143*, 29–36.
12. Hoffman RM. Screening for Prostate Cancer *N Engl J Med* **2011**, *365*, 2013–2019.
13. Lilja, H. A kallikrein-like serine protease in prostatic fluid cleaves the predominant seminal vesicle protein. *J. Clin. Invest.* **1985**, *76*, 1899–1903
14. Logozzi, M.; De Milito, A.; Lugini, L.; Borghi, M.; Calabrò, L.; Spada, M.; Perdicchio, M.; Marino, M.L.; Federici, C.; Iessi, E.; et al. High levels of exosomes expressing CD63 and caveolin−1 in plasma of melanoma patients. *PLoS One.* 2009, 4, e5219, doi:10.1371/journal.pone.0005219.
15. Logozzi, M. Angelini D.F.; Iessi, E.; Mizzoni, D. Di Raimo, R.; Federici, C.; Lugini, L.; Borsellino, G.; Gentilucci, A.; Pierella, F.; Marzio, V.; Sciarra, A.; Battistini, L.; Fais, S. Increased PSA expression on prostate cancer exosomes in in vitro condition and in cancer patients. *Cancer Lett.* **2017**, *10*, 318–329.
16. Logozzi, M.; Mizzoni, D. Angelini D.F. Di Raimo, R.; Falchi, M.; Battistini, L.; Fais, S. Microenvironmental pH and Exosome Levels Interplay in Human Cancer Cell Lines of Different Histotypes. *Cancers.* **2018**, *5*, 10, doi:10.3390/cancers10100370.
17. Parolini, I.; Federici, C.; Raggi, C.; Lugini, L.; Palleschi, S. De Milito, A.; Coscia, C.; Iessi, E.; Logozzi, M.; Molinari, A.; Colone, M.; Tatti, M.; Sargiacomo, M.; Fais, S.Microenvironmental pH is a key factor for exosome traffic in tumor cells. *J. Biol. Chem*. **2009**, *4*, 34211–34222.
18. Schroder, F.H.; Hugosson, J. Roobol, M.J.; et al. Screening and prostate-cancer mortality in a randomized European study. *N. Engl. J. Med.* **2009**, *360*, 1320–1328.
19. Shah, R.; Patel, T.; Freedman, J.E. Circulating Extracellular Vesicles in Human Disease. *N. Engl. J. Med.* **2018**, *379*, 2180–2181.
20. Taichman, R.S.; Loberg, R.D.; Mehra, R.; Pienta, K.J. The evolving biology and treatment of prostate cancer. *J. Clin. Invest*. **2007**, *117*, 2351–2361.
21. Théry, C.; Witwer, K.W.; Aikawa, E.; Alcaraz, M.J.; Anderson, J.D.; Andriantsitohaina, R.; Antoniou, A.; Arab, T.; Archer, F.; Atkin-Smith, G.K.;et al. Minimal information for studies of extracellular vesicles 2018 (MISEV2018): a position statement of the International Society for Extracellular Vesicles and update of the MISEV2014 guidelines. *J. Extracell. Vesicles*. **2018**, *7*, 1535750, doi:10.1080/20013078.2018.1535750.
22. Turchinovich, A.; Drapkina, O.; Tonevitsky, A. Transcriptome of Extracellular Vesicles: State-of-the-Art. *Front. Immunol.* **2019**, *28*, 202.
23. van Niel, G. D'Angelo, G.; Raposo, G. Shedding light on the cell biology of extracellular vesicles. *Nat Rev Mol. Cell. Biol.* **2018**, *19*, 213–228.
24. Yáñez-Mó, M.; Siljander, P.R.; Andreu, Z.; Zavec, A.B.; Borràs, F.E.; Buzas, E.I.; Buzas, K.; Casal, E.; Cappello, F.; Carvalho, J.; et al. Biological properties of extracellular vesicles and their physiological functions. *J. Extracell. Vesicles.* **2015**, *14*, 27066, doi: 10.3402/jev.v4.27066.

https://www.cancer.gov/types/prostate/psa-fact-sheet

https://gco.iarc.fr/today/data/factsheets/cancers/27-Prostate-fact-sheet.pdf

**Ethical Committee Approval**

*Comitato Etico*
*Istituto Superiore di Sanità*
*Viale Regina Elena 299*
*00161 Roma*

Prot. PRE-275/17

Presidente
Prof. Walter Ricciardi
Istituto Superiore di Sanità
Sede

Dott. Stefano Fais
Dip. OMM
Sede

**Oggetto**: Comunicazioni relative alle modifiche apportate al progetto "Espressione di PSA su esosomi plasmatici di pazienti affetti da ipertrofia prostatica benigna e da carcinoma della prostata: Individuazione di un cut-off per studi di popolazione". Responsabile Dott. Stefano Fais

Su convocazione del Presidente, il Comitato Etico si è riunito il giorno 6 giugno 2017.
Membri presenti: Amadori, Corgatelli, Daniele, Evangelista, Iardino, Malchiodi-Albedi, Petrini, Pichini, Popoli, Rezza, Silano, Salvatore, Tartaglia, Ugazio.
Membri assenti: Fiori, Galletti, Gambino, Guarino, Pintor, Pocchiari.

Il progetto è stato approvato dal Comitato Etico (seduta del 18 aprile 2017, Rif. Prot. PRE-275/17) con richiesta di modifiche al consenso informato e al protocollo di studio. Esaminata la nuova documentazione, il comitato etico approva la sperimentazione in via definitiva, con il suggerimento di adottare come consenso informato il testo allegato revisionato dal Comitato Etico.
Il parere è espresso all'unanimità.

Roma, 6 giugno 2017

Il Presidente
del Comitato Etico dell'ISS

© 2019 by the authors. Licensee MDPI, Basel, Switzerland. This article is an open access article distributed under the terms and conditions of the Creative Commons Attribution (CC BY) license (http://creativecommons.org/licenses/by/4.0/).
